# Social reward and support effects on exercise experiences and performance: Evidence from parkrun

**DOI:** 10.1371/journal.pone.0256546

**Published:** 2021-09-15

**Authors:** Arran J. Davis, Pádraig MacCarron, Emma Cohen

**Affiliations:** 1 Social Body Lab, Institute of Human Sciences, University of Oxford, Oxford, United Kingdom; 2 Mathematics Applications Consortium for Science and Industry, University of Limerick, Limerick, Ireland; 3 Wadham College, Oxford, United Kingdom; University of Pavia: Universita degli Studi di Pavia, ITALY

## Abstract

There is growing academic, civic and policy interest in the public health benefits of community-based exercise events. Shifting the emphasis from competitive sport to communal activity, these events have wide appeal. In addition to physical health benefits, regular participation can reduce social isolation and loneliness through opportunities for social connection. Taking a broad evolutionary and social psychological perspective, we suggest that social factors warrant more attention in current approaches to physical (in)activity and exercise behavior. We develop and test the hypothesis that social reward and support in exercise are associated with positive exercise experiences and greater performance outputs. Using a repeated-measures design, we examine the influence of social perceptions and behavior on subjective enjoyment, energy, fatigue, effort, and objective performance (run times) among a UK sample of parkrun participants. Social factors were associated with greater subjective enjoyment and energy. Higher subjective energy, in turn, was associated with faster run times, without any corresponding increase in perceived effort. No significant main effects of social factors on fatigue, performance or effort were detected. The role of social structural factors has long been recognized in public health approaches to physical activity. Our results indicate that there should be greater research attention on how positive and rewarding social behaviors and experiences—particularly subjective enjoyment and energy, and perceptions of community social support and belonging—influence exercise-related behavior, psychology and physiology, and promote health through collective physical activity. The research also supplements traditional emphases on social facilitation and team sport that have dominated sport and exercise psychology and offers new avenues for understanding the deep connections among psychological, social and physical function in everyday health.

## Introduction

Physical activity and social relationships are critical “flashpoints” for health policy [1, 2]. Low levels of physical activity and high levels of loneliness have been independently associated with poorer mental and physical health outcomes and mortality [3]. Despite the established benefits of sufficient physical activity and secure social ties for mental and physical health, levels of physical inactivity are extremely high globally [e.g., 4, 5]. Although evidence on the prevalence of loneliness is less well established, loneliness as the *perception* of social isolation (even when among other people), is also widely recognized as a “real mental health challenge for the nations” [6] and a growing problem worldwide [7, 8].

Medicalist perspectives predominate in assessments of the negative effects of physical inactivity and loneliness (e.g., morbidity-mortality risks, “pandemic” terminology). Nevertheless, these are widely considered to be complex social issues that require collaborative, integrated and holistic public health approaches [9]. Evidence suggests that one problem compounds the other, with loneliness having been identified as a risk factor for physical inactivity [10, 11] and physical inactivity as a risk factor for psychiatric and psychosocial health problems that are directly or indirectly associated with depression and loneliness [12, 13]. Physical inactivity and loneliness can therefore be approached as interlinked problems that can be jointly addressed via independent interventions, but that can also benefit from integrated solutions. Toward this general aim, this paper contributes new theoretical and empirical perspectives on the behavioral and psychological synergies between physical exercise and social relationships.

Although considerable research attention has been directed at understanding how social-environmental factors influence physical activity behavior [e.g., 14], connections between the affective dimensions of physical activity, particularly exercise, and sociality remain underappreciated. Previous research offers some promising clues. For example, positive affect in exercise is a key determinant of adherence [15] and, in general, intense emotional experiences happen more often in the context of interdependent social connection and belonging than in independent situations [16]. This suggests a possibility for social enhancement of positive affect in physical exercise [17–20], with corresponding increases in motivation and adherence. In addition, there are links between collective physical activity in diverse forms, such as play, sport, dance, and exercise, and feelings of social bonding and belonging [e.g., 18, 21], which in turn are associated with positive motivation and adherence [22]. Taken together, these links trace a virtuous circle between affectively rich, meaningful and rewarding social connections and intrinsically motivated engagement and enjoyment in collective physical activity.

Besides links to social-motivational psychology, there are effects of perceived social support on the homeostatic regulation of stress, fatigue and pain [e.g., 23–25]. Moreover, rewarding social experiences entail activation of endogenous neurobiological systems, such as the opioidergic and endocannabinoid systems [26–28], that are also involved in modulating responses to nociceptive stimuli and in sustaining endurance exercise [29–32]. Applying these insights, it can be hypothesized that social support and social reward buffer, or reduce, unpleasant exercise-induced affect, such as fatigue and pain [17], and boost feelings of enjoyment, thereby potentially increasing performance outputs, sense of achievement, and engagement in exercise. Performance improvements in this case are not necessarily a consequence of increased goal-directed motivation, but rather reduced subjective effort or increased subjective energy [33].

Despite the apparent connections among affective and behavioral dimensions of exercise and sociality, surprisingly little research has directly investigated the effects of either the rewarding or buffering aspects of social bonding and support on exercise experiences. Traditional social psychological approaches in sports and exercise science have focused on competitive and evaluative aspects of social presence and their facilitating effects on motivation, effort and performance across different types of task [for a review, see 34]. In a largely separate line of research, team cohesion has been studied as a predictor of effort and performance in sport. For example, group cohesion in sport settings—defined as “a group dynamic process that is reflected in the tendency of a group to stick together and remain united in the pursuit of instrumental objectives and/or for the satisfaction of member affective states” [35; p. 213]–positively predicts performance success [35], adherence to group exercise programs [36] and physical exertion in team sports [37]. Related research drawing inspiration from social identity theory has begun to identify the importance of social-group identities for promoting physical activity engagement, adherence, enjoyment and effort. For example, in a recent parkrun study, Stevens et al. [38] found that stronger identification with the parkrun running group positively predicted participation, life satisfaction, exercise-specific satisfaction, and group cohesion. These and other studies [39, 40] offer support for the idea that individuals’ perceptions of the social group as cohesive and supportive, and with which they can strongly identify as group members, can promote positive affective exercise experiences, increase participation in physical activity, and facilitate performance via socially mediated mechanisms other than arousal, evaluation apprehension and distraction.

This literature has elucidated the types, causes and consequences of group cohesion in physical exercise, predominantly in team contexts. However, the affective and performance effects of social reward and support, particularly in transient collectives, virtual settings, and exercise groups without clearly defined boundaries, interdependent roles and shared goals, remain relatively unexplored, both theoretically and empirically, and little is known about the psychobiological pathways via which human sociality, psychology and biology co-regulate one another in exertive physical activity [17, 19].

Preliminary experimental evidence suggests that exercising with others versus alone leads to significantly greater pain thresholds, and cues to social bonding prior to exercise improve subsequent performance outputs, with no corresponding increase in subjective fatigue [17, 19, 41]. Here, we build on these findings to investigate how social reward and perceived support modulate positive affect, feelings of energy and fatigue, and performance in the context of *parkrun*, a community-based organization that convenes free, weekly, timed 5 km runs in public parks and spaces. Parkrun offers a suitable naturalistic setting in which to study associations among social experiences and exercise. The aim of parkrun is to “promote physical activity and community spirit, by providing supportive opportunities to exercise” [42; p. 171]. According to a study conducted at one UK parkrun site, ‘social togetherness’ was the second most important aspect of parkrun among surveyed participants, following “getting exercise” [43]. Since its inception in 2004, parkrun has seen rapid and sustained international growth; at the time of writing, parkrun events occur in over 2,000 locations in 22 countries worldwide. Using ecologically valid measures in this naturalistic setting can provide much-needed insight into the appeal and public health value of such community-based initiatives [44].

In the current study, we used survey data to investigate effects of three predictor variables on feelings of enjoyment, fatigue, and energy as well as objective performance among parkrunners. The three predictor variables aimed to capture behaviors and subjective assessments associated with social reward, or positive and enjoyable social interactions [45], and support in an ecologically valid way: 1) whether participants attended with friends or family vs. attended alone; 2) whether or not participants interacted with others socially before the event; 3) the degree to which participants felt a) supported by, and b) integrated into the parkrun community. Hypotheses are summarized in Table 1.

**Table 1:** Research hypotheses.

	**H1. Main effects of social predictors on subjective fatigue [e.g**., 17**]**
**1.1–1.3**	Higher subjective ratings of community support and integration will predict lower fatigue (1.1); coming or meeting up with friends/family will predict lower fatigue (1.2); being social (vs. not being social) before the run will predict lower fatigue (1.3).
	**H2. Main effects of social predictors on subjective energy [e.g**., 46**]**
**2.1–2.3**	Higher subjective ratings of community support and integration will predict higher energy (2.1); coming or meeting up with friends/family will predict higher energy (2.2); being social (vs. not being social) before the run will predict higher energy (2.3).
	**H3. Main effects of social predictors on subjective enjoyment [e.g**., 42**]**
**3.1–3.3**	Higher subjective ratings of community support and integration will predict higher enjoyment (3.1); coming or meeting up with friends/family will predict higher enjoyment (3.2); being social (vs. not being social) before the run will predict higher enjoyment (3.3).
	**H4. Main effects of social predictors on 5 km run times [e.g**., 17**]**
**4.1–4.3**	Higher subjective ratings of community support and integration will predict faster 5 km run times (4.1); coming or meeting up with friends/family will predict faster 5 km run times (4.2); being social (vs. not being social) before the run will predict faster 5 km run times (4.3).
	**H5 & H6. Mediators of main effects of social predictors on 5 km run times**
**5.1–5.3**	Higher scores on the social predictor variables (5.1: community support and integration | 5.2: coming or meeting up with friends/family | 5.3: pre-run sociality) will predict higher perceived energy levels, and higher perceived energy levels will predict faster 5 km run times.
**6.1–6.3**	Higher scores on the social predictor variables (6.1: community support and integration | 6.2: coming or meeting up with friends/family | 6.3: pre-run sociality) will predict lower fatigue, and lower fatigue will predict faster 5 km run times.

## Materials and methods

### Participants

Participants were recruited in person and through parkrun event webpages from six parkrun sites in southern England. Sites were selected for their proximity to Oxford to enable us to recruit participants in person at the events and were agreed upon with the parkrun Research Board. Recruitment was on a rolling basis over approximately two months. Participants were required to be at least 18 years old. There were no other inclusion or exclusion criteria. See S1 SOM of 1 for further description of the parkrun research context.

Surveys were administered online via Qualtrics, and participants were given the option of receiving the link to the online survey by email, text or mail (no participants chose mail). All parkruns are held weekly, on Saturdays at 9:00am. Survey links were sent at 09:45 on every Saturday for the duration of the study and participants were requested to complete the survey as soon as possible after their run. Participants were encouraged to attend parkrun as usual during the study period and to respond to the survey when they did so, although it was made clear that they were not required to respond to the survey each time they attended a parkrun (this met the request of the parkrun Research Board that the study be minimally intrusive; see S1 SOM of 2 for more details on survey recruitment and study dates). In total, 188 parkrunners consented to take part; 144 participants completed the survey at least once and there were 734 usable surveys in total. This study was approved by the parkrun Research Board (UK) and the School of Anthropology and Museum Ethnography Departmental Research Ethics Committee, University of Oxford (reference number: SSH_SAME_C1A_15_084) and all participants gave prior informed consent. Data collection did not continue after data analysis.

### Survey procedure

The survey consisted of 12 questions concerning participant motivations, perceptions of community support and integration and other social aspects of their run, and about fatigue, pain, and effort. Participants were first asked: “Besides other motivations you might have had for attending parkrun today, which of the following options best applies to you? I was motivated to… (a) improve my ranking; (b) improve my time; (c) run together with other people.” Only one response option was permitted. The remaining eleven questions were asked in a randomized order. On seven-point Likert Scales (1—not at all, 7—very much), participants responded to the questions: “How much did you feel supported by the parkrun community today?”, “How much did you feel you were a part of the parkrun community today?” These two questions were later combined using principal components analysis (PCA) in a single ‘parkrun community component’. These items were derived specifically for this survey.

Participants also used seven-point Likert Scales (1—*not at all*, 7—*very much*) to respond to the questions: “How much did you enjoy your run today?”, “How energizing did it feel to be with the other parkrunners today?”, and “How physically fatigued did you feel during your run today?” (if participants answered with a 5 or greater on the seven-point Likert Scale for this question, they were immediately asked, using the same scale, “How physically painful did this fatigue feel?”). Questions about energy and fatigue levels were adapted from the Profile of Mood States items measuring ‘Vigor-Activity’ and ‘Fatigue-Inertia’ [47, 48]. As a measure of their effort, participants were also asked: “Please rate your feeling of exertion (how much physical effort you felt you were giving) during your run today”, with response options following the Borg Rating of Perceived Exertion scale [49] (see S1 SOM of S1 Fig).

Participants were also asked about social aspects of their run: “Please choose the answer that best describes your run today. Today I ran… (a) on my own; (b) alongside one or more acquaintances; (c) alongside one or more friends/family members; (d) alongside a mix of acquaintances and friends/family members”. Two questions asked participants about their sociality before the run: “Please choose the option that best describes what you were doing just before you went to the start line today; (a) Getting ready and hanging out on my own; (b) Getting ready and hanging out with others; (c) Something else (e.g., rushing to get the start line, chatting on my phone, etc.)”, and “Did you come along with, or meet up with, anyone else at parkrun today? Please choose the answer that best applies to you. Today I came/met up with… (a) nobody else; (b) one or more acquaintances; (c) one or more friends/family members or a mix of acquaintances and friends/family members.”

To assess the extent to which participants’ running pace was influenced by running with others, they were further asked: “Which of the following best applies to you? (a) Today I slowed down for my running partner(s); (b) Today I sped up for my running partner(s); (c) Today my natural pace was pretty much the same as the pace of my running partner(s); (d) Not applicable—I ran on my own.”

### Additional data acquisition

Each parkrun event location has its own page within the parkrun website (e.g., https://www.parkrun.org.uk/abingdon/). These websites are used to communicate with participants and to report event results; the results of each parkrun event are posted on a ‘results’ page that contains information on event participants’ 5 km run time, ranking, gender, age category, and running club. Participants’ 5 km run times, gender, and age category were collected from these parkrun online databases for every run for which a survey response was recorded. Survey responses were linked to event data (run times) using the event date and parkrun ID numbers provided in each survey (see S1 SOM of 3 for full details on additional data acquisition).

## Analyses

### Inferential statistical models

The survey data consisted of repeated measures nested within individuals—each participant had one or more survey responses. Multilevel modelling was thus required to analyze the data. All multilevel models included participant ID as the level-two grouping variable. When possible, maximal random effects structures were used. All models included random slopes for all predictor variables, unless the inclusion of a random slope caused the model to fail to converge, in which case the random slope term was removed from the model (see S1 SOM of 4).

For the multilevel models testing effects of social predictor variables on subjective fatigue, energy, and enjoyment (Hypotheses 1.1–3.3), there were no covariates. Models on 5 km run times (logged to improve model fit) and mediation analyses included a ‘pace influence’ covariate, which quantified the degree to which participants’ adjusted their running pace to running alongside others (i.e., faster, slower, no change). All direct, indirect, and total effects were calculated following the procedures of Tingely et al. [50]. Additional models examined effects of response frequency and response time on self-reported outcome variables and of social predictors on effort.

Statistical analyses were performed in R version 3.5.3. The R packages *lme4* [51] and *lmer* [52] were used to perform the multilevel modelling. Marginal *R*^*2*^ ($R_{m}^{2}$ and $R_{c}^{2}$) for the multilevel models was calculated using functions from the *piecewiseSEM* package in R [53]. The R packages *mediation* [50] and *lme4* [51] were used to perform the multilevel mediation analyses, which employed bias-corrected and accelerated (BCa) bootstrap-based confidence intervals.

### Family-wise error rates

When testing families of comparisons, it is necessary to control for the increased probability of Type I error due to conducting multiple hypothesis tests [54–56]. The analyses reported below used the procedures of Benjamini and Hochberg [54] in determining more conservative critical values for the multiple comparisons over the three related tests in each of the six families of hypotheses in Table 1. The full procedure used for controlling for multiple comparisons can be found in S1 SOM of 5.

For the mediation analyses, each type of effect (indirect, direct, and total) was treated as a sub-family of tests when accounting for multiple comparisons for Hypothesis 5.1—Hypothesis 5.3 and Hypothesis 6.1—Hypothesis 6.3.

### Creation of social predictor variables

For the inferential analyses reported below, three social predictor variables were derived from the self-report survey items on participants’ sociality at parkrun. We created binary categories for the items on who participants came/met up with and pre-run sociality. Regarding who participants came or met up with, we combined responses into either (1) coming/meeting up with friends and/or family, or (2) coming/meeting up with one or more acquaintances or coming on their own. Regarding pre-run sociality we combined responses into either (1) being social before the run (‘getting ready and hanging out with others’), or (2) not being social before the run (‘getting ready and hanging out on my own’ or ‘something else’). Responses to the two questions on the parkrun community were highly correlated (“How much did you feel supported by the parkrun community today?” and “How much did you feel you were a part of the parkrun community today?”; *r* = .72). A PCA was used to test the relationship between these two variables, based on the expectation that the questions on support and inclusion would load onto a component related to social support from the parkrun community (see S1 SOM of 6 for a full summary of this analysis). The single component extracted from the two variables had a Kaiser’s criterion of 1.72, explained 86% of the variance in answers to the two questions, and had good reliability (Cronbach’s alpha = .840). The component (henceforth, the ‘parkrun community component’) was taken to represent the perceived strength of participants’ relationship with the parkrun community. These three predictor variables (co-participation, pre-run sociality, community) were used in the analyses described below.

## Results

### Survey responses

In total, there were 765 survey responses. Some returns could not be matched to a particular parkrun ID and were excluded from analyses. If a participant responded more than once for a given event, their first response was retained for analysis, and all subsequent responses for that event were removed (see S1 SOM of 3 for full data cleaning procedures). After exclusions, there were 734 survey responses from 143 participants; 49% of participants were female (*n* = 70), and females represented 46% of all surveys returns analyzed (*n* = 341). Respondents were drawn from all age categories (18–20–75–79). The mean age of survey respondents (taking the midpoint of the age category as the respondent’s age) was 48.27 years (median = 47 years, *SD* = 11.90 years).

Participants responded to the survey an average of 5.09 times (median = 4, *SD* = 4.02, range = 1–17; see S1 SOM of S2 Fig for a histogram of participant’s total survey responses and S1 SOM of S3 Fig for histograms of survey return and completion times). Thirty-four participants (22%) were responsible for 50% of all survey responses (n = 369); see S1 SOM of 7 and S1 Table for summaries of differences between scores on the predictor, mediator, and outcome variables of interest for respondents with high and low response counts. The median survey response time (measured from the time participants clicked on the link to the survey to the time they submitted their final answer) was 4 hr 32 min. Survey response times did not affect responses to affect related questions; logged (to improve model fits) response times did not predict subjective fatigue, perceived energy, subjective enjoyment, or the parkrun community component (see S1 SOM of 8 and S2–S5 Tables).

### Descriptive results

Descriptive results for survey and run time data are summarized in Table 2. Participants were most often motivated to attend parkrun for social reasons, i.e., “to run together with other people” versus attending for reasons related to training (“to improve my time) or competition (“to improve my ranking”). Participants most often reported coming or meeting up with friends and family, or a mixture of friends, family, and acquaintances, and socializing with others before the run (versus being alone or doing something else). Perceived support and integration into the parkrun community was relatively high overall, as were mean enjoyment and energy.

**Table 2:** Descriptive results for survey and run-time data.

**Panel A. Frequency Data (% of survey returns)**		
**Measure**	**Response**	**% (n) of survey responses**
*Motivation*	Social	61.04 (448)
	Training	36.51 (268)
	Competition	2.45 (18)
*Co-running*	Solo	67.71 (497)
	Friends / family	14.44 (106)
	Acquaintances	11.58 (85)
	Friends / family / acquaintances	6.40 (47)
*Pace Influence*	Slowed down	8.17 (60)
	Sped up	6.13 (45)
	Natural pace	11.44 (84)
	Not applicable (running alone)	74.25 (545)
*Co-participation*	Solo	18.80 (138)
	Acquaintances	27.52 (202)
	Friends / family / acquaintances	53.68 (394)
*Pre-run Sociality*	Solo	25.75 (189)
	Social	62.94 (462)
	Other	11.31 (83)
**Panel B. Mean Data**		
**Measure (response scale)**	**Mean (SD; range)**	
*Parkrun community*:		
*Support (1–7)*	5.72 (1.06; 2–7)	
*Integration (1–7)*	5.76 (1.08; 1–7)	
*Effort (6–20)*	14.61 (2.25, 6–20)	
*Fatigue (1–7)*	4.79 (1.27; 1–7)	
*Pain (1–7; 64*.*17%*, *n = 471)*	4.24 (1.32, 1–7)	
*Enjoyment (1–7)*	5.64 (1.12, 1–7)	
*Energy (1–7)*	5.64 (1.06; 1–7)	
*Run time (min*:*sec)*	27:52 (5:53; 16:36–57:20)	

Although attending or meeting up with others appears to be the norm for this sample, the majority of survey responses indicate that participants ran on their own. Regarding the question on who participants ran with (henceforth the ‘co-running’ variable), 67.71% of returns indicate that the participant ran alone. Regarding the question on whether participants changed their running pace to run with a running partner (henceforth the ‘pace influence’ variable), 74.25% of returns indicate that the participant ran alone. This difference in proportions likely results from the different phrasing of the two questions. For example, it is possible that some participants ran alongside multiple different others during the event (as captured by the co-running measure), but that they did not run together with (or therefore coordinate pace with) particular individuals (as captured by the pace influence measure).

Regarding subjective effort, the mean response on the perceived effort scale was 14.61, between “13—Somewhat hard” and “15—Hard (heavy)”. Subjective effort was highly correlated with subjective fatigue (*r* = .706). The follow-up to the question on fatigue, on how physically painful the fatigue was, was only asked when participants’ reported level of fatigue was 5 or greater (64.17% of surveys; *n* = 471), and was slightly lower than the overall level of fatigue. Participants’ 5 km run times were positively skewed (skewness = 0.87; see S1 SOM of S4 Fig). Although informative of the survey results as a whole, this descriptive picture should be interpreted with caution as it is most heavily influenced by those participants who answered the survey the most times (see S1 SOM of S2 Fig).

### Main effects of social predictor variables on subjective experiences and 5km run times

#### Main effects of social predictor variables on subjective fatigue (H1)

The parkrun community component and pre-run sociality did not significantly predict participants’ perceptions of fatigue (see S1 SOM of S5 and S6 Tables). There was an association between who participants came/met up and perceptions of fatigue; coming/meeting up with friends/family (vs. coming or meeting up with acquaintances or alone) predicted significantly lower perceptions of fatigue, *b* = −0.250, *SE* = 0.111, *p* = .027 (see S1 SOM of S8 Table), but this *p*-value was not significant after adjustment for multiple comparisons.

#### Main effects of social predictor variables on perceived energy (H2)

The parkrun community component positively predicted perceived energy, *b* = 0.559, *SE* = 0.034, *p* < .001, as did coming or meeting up with friends/family, *b* = 0.209, *SE* = 0.081, *p* = .009, and pre-run sociality, *b* = 0.310, *SE* = 0.092, *p* = .001 (see S1 SOM of S9–S11 Tables). All *p*-values were significant after adjustment for multiple comparisons.

#### Main effects of social predictor variables on subjective enjoyment (H3)

The parkrun community component positively predicted enjoyment, *b* = 0.439, *SE* = 0.039, *p* < .001, as did coming or meeting up with friends/family, *b* = 0.230 *SE* = 0.086, *p* = .008, and pre-run sociality, *b* = 0.346, *p* < .001 (see S1 SOM of S12–S14 Tables). All *p*-values were significant after adjustment for multiple comparisons.

#### Main effects of social predictor variables on 5 km run times (H4)

None of the social predictor variables predicted (logged) 5 km run times (see S1 SOM of S15–S17 Tables).

#### Perceived energy as a mediator of main effect of social predictors on 5 km run times (H5)

**Fig 1:**
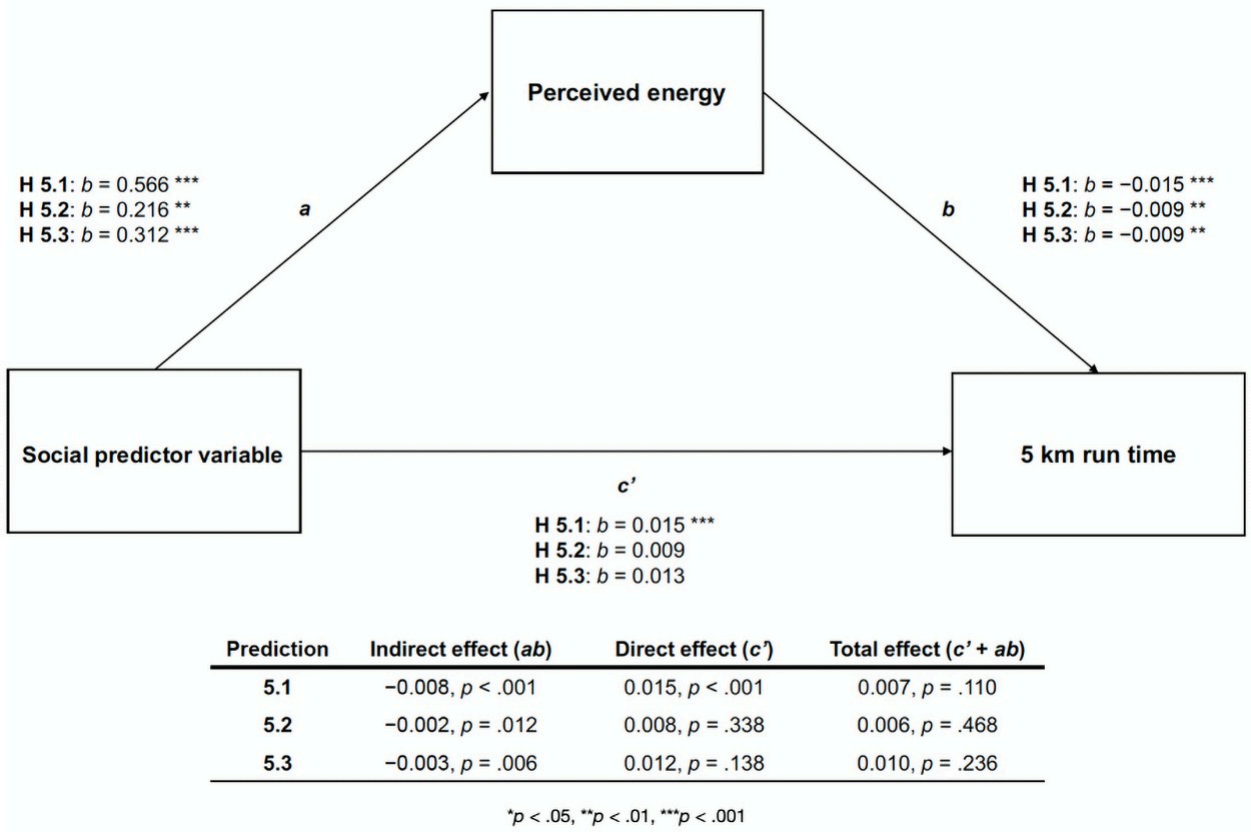
Mediation of social predictors on 5km run times. Mediation diagram depicting the direct, indirect, and total effects of the social predictor variables—the parkrun community component (H5.1), whether or not participants came or met up with family and/or friends (H5.2), and their pre-run sociality (H5.3)–on 5 km run times, with participants’ perceived energy as a potential mediator.

All three predictor variables had significant, ergogenic indirect effects on 5 km run times, with perceived energy as the mediating variable (H5.1–5.3; see Fig 1 and S1 SOM of S5 Fig).

The first mediation analysis tested whether participants’ perceived energy mediated the relationship between their scores on the parkrun community component and their (logged) 5 km run times (5.1). Analyses revealed a significant average indirect effect of −0.008 (*p* < .001), a significant average direct effect of 0.015 (*p* < .001), and a non-significant total effect of 0.007 (*p* = .110): how energized participants felt mediated the relationship between the parkrun community component and participants’ 5 km run times. The average indirect effect was significant after adjustment for multiple comparisons (see S1 SOM of S18 and S19 Tables for model summaries and S1 SOM of S5a and S5d Fig for predictor, mediator, and outcome variable relationships).

The second mediation analysis tested whether participants’ perceived energy mediated the relationship between who participants came or met up with and their (logged) 5 km run times (H5.2). There was a significant average indirect effect of −0.002 (*p* = .012), a non-significant average direct effect of 0.008 (*p* = .338), and a non-significant total effect of −0.006 (*p* = .468): the relationship between who participants came or met up with and logged run times was mediated by how energized they felt. The average indirect effect was significant after adjustment for multiple comparisons (see S1 SOM of S20 and S21 Tables for model summaries and S1 SOM of S5b and S5d Fig for predictor, mediator, and outcome variable relationships).

The third mediation analysis tested whether participants’ perceived energy mediated the relationship between their pre-run sociality and their (logged) 5 km run times (H5.3). Results showed a significant average indirect effect of −0.003 (*p* = .006), a non-significant average direct effect of 0.012 (*p* = .138), and a non-significant total effect of 0.010 (*p* = .236): the relationship between participants’ pre-run sociality and logged 5 km run times was mediated by how energized they felt. The average indirect effect was significant after adjustment for multiple comparisons (see S1 SOM of S22 and S23 Tables for model summaries and S1 SOM of S5c and S5d Fig for predictor, mediator, and outcome variable relationships).

#### Subjective fatigue as a mediator of main effect of social predictors on 5 km run times (H6)

With subjective fatigue as the mediating variable (H6), none of the social predictor variables had significant direct, indirect, or total effects on 5 km run times. Mediation summaries can be found in S1 SOM of S6 Fig, and model summaries can be found in S1 SOM of S24–S29 Tables.

### Main effects of social predictor variables on subjective effort

None of the social predictor variables significantly predicted subjective effort levels (see S1 SOM of S29–S32 Tables). This is consistent with the claim that the social predictor variable effects on participant experiences and performance are not confounded by social-motivational factors associated with competition or threat of evaluation.

### Model assumption checks

Assumption checks were carried out in accordance with previously published methods [57, 58]. Multilevel model assumptions include residual homoscedasticity, normality, and linearity (at both level-one and level-two of the model); the assumptions of multilevel mediation models are the same, but also include those of sequential ignorability [59]. Model assumption checks and are reported in S1 SOM of 9.

## Discussion

Community-based sports and exercise events, particularly those offered on a continuous and regular basis, have been identified as having significant but untapped public health benefits [60]. Existing evidence suggests that participation in collective exercise events, such as parkrun, can be encouraged not just for the physical and psychological benefits of physical activity and exercise, but also for the simultaneous wellbeing benefits of social connection, integration and support [22, 61]. Although it has been shown that individuals often draw on existing connections to initiate their participation in parkrun [e.g., 62], and that stronger identification with the parkrun collective is associated with more frequent participation and higher life satisfaction [38], little is known about the effects of social reward and support on participants’ affective experiences of exercise, and related performance. Addressing this gap can begin to contribute valuable new data relevant to tackling pressing international public health challenges of physical inactivity and loneliness, while at the same time advancing our scientific understanding of the social modulation of homeostatic mechanisms that contribute to feelings of fatigue, energy, enjoyment and performance in exertive physical activity [63].

To investigate the social determinants of affective experiences and performance in exercise in a naturalistic group setting, we analyzed associations between a range of social variables and self-reported enjoyment, fatigue and energy, as well as recorded run times, in a sample of UK parkrunners. Results give partial support to our hypotheses and suggest a more nuanced account of how social environments affect experiences and outputs during physical exercise.

Survey responses confirmed previous findings of high levels of sociality at parkrun [42, 43, 64]: in the majority of surveys, participants reported coming or meeting up with friends and family, or a mixture of friends, family, and acquaintances. Running with others, rather than running to improve times or rankings, was the predominant motivation for attending. As participants primarily report running on their own, however, this motivation appears to relate more to the parkrun collective rather than specific running partners or groups. Perceived support from and integration within the parkrun community was high, as were subjective enjoyment, energy, effort and fatigue. Overall, the descriptive picture is of a positive and facilitative social context for invigorating and challenging self-paced exercise.

Social predictors (coming or meeting up with friends and/or family vs. coming alone; hanging out with others before the event vs. alone; feeling integrated into and supported by the parkrun community) had positive effects on subjective enjoyment and energy, and performance, in line with our hypotheses. The social predictor variables did not directly predict participants’ perceptions of fatigue, however, and it was only through indirect effects that they were associated with lower 5 km run times. Specifically, all of the social predictor variables had significant indirect negative effects on 5 km run times, via positive effects on subjective energy. A one unit increase in the social predictor variables led to a decrease between 0.17% and 0.74% (depending on the predictor variable) in 5 km run times, such that a one unit increase in the social predictor variables led to between 3.34 s and 11.70 s faster 5 km run times, on average [65]. Taken together, these results suggest that, even among those who generally run slower times, it is not social factors *per se* that lead to improved performance. Rather, social factors, on average, are positively associated with greater feelings of energy, and these predict faster 5 km run times. In fact, when controlling for subjective energy, there was a significant direct effect of the parkrun community factor on run times, but in the opposite direction predicted. This likely reflects the variable performance motivations represented across these participants; those with stronger motivations to improve their run times may be more likely to benefit from the energizing effects of social support and integration relative to those less concerned about their running performance.

Overall, these results point to potential beneficial effects of social reward and perceived social support on positive affect, including enjoyment and subjective energy, with potential regulatory effects on performance. As there is no indication that social predictors were associated with self-reported effort, it seems unlikely that the mediating effect of subjective energy on performance is attributable to motivations traditionally identified in social facilitation research (e.g., apprehension about being evaluated). Rather, we suggest that the felt energy and enjoyment associated with the social predictor variables can be explained, in part, by the intrinsic psychological reward of positive and supportive social engagement [66, 67]. This interpretation is in line with observational research showing that perceptions of social support and cohesion at parkrun are associated with more positive experiences [68]. Although this study does not examine potential causal mechanisms, our interpretation is also corroborated by extensive neurobiological evidence that endogenous systems involved in sustaining physical exercise, such as the endocannabinoid and opioidergic systems, are also activated by positive social interactions [29, 67, 69–72]. Overall, we suggest that positive social engagement and perceptions of support modulate the balance of pleasure-displeasure that regulates self-selected exercise intensity and performance output, and that this could be instrumental in motivating adherence [15, 63, 73].

Although all of the social predictor variables were positively associated with participants’ subjective energy, there was no significant relationship detected between the social predictors and fatigue, nor any mediation effect of social predictors and fatigue on performance. These results suggest a qualification of our account. Fatigue and energy are both conceptualized in the literature as multidimensional states that concern ability to sustain voluntary activity. Whereas feelings of fatigue relate specifically to the perceived *difficulty* of maintaining task goals [74], feelings of energy relate to perceived *ability* to maintain task goals, captured in standard measures as “vigor” or “vitality”. Both reported fatigue and reported energy were relatively high (*M* = 4.79, *SD* = 1.27 and *M* = 5.64, *SD* = 1.06, respectively, on a 1–7 scale). Our results suggest a distinction between “boosting” (i.e., energy-giving) and “buffering” (i.e., fatigue-reducing) mechanisms and effects on affect and related performance. All three social predictor variables showed similar effects on outcome variables. Future research could systematically manipulate those aspects of social behavior most directly associated with increasing pleasant affective states, such as laughter, behavioral synchrony [e.g., music, singing or dancing; 75] and those most directly associated decreasing unpleasant affective states [e.g., social cues of safety/support; 76], and measure effects on subjective energy and fatigue.

An alternative explanation for the significant mediation effect is that the presence of known social others increased levels of competition and motivation among parkrunners, and these effects were captured by the perceived energy variable. Motivation and felt energy are likely overlapping constructs, both leading to increased physical outputs [77]. It is possible that the presence of friends or family members increases competition levels at parkrun. However, it is unclear why socializing before the event or feeling more included and supported by the parkrun community would predict higher competition levels. Furthermore, contrary to the competitive-motivation account, we found no association between social predictor variables and subjective effort. Future studies could investigate relationships among social reward or support, felt energy and fatigue, and performance in a range of social exercise settings. Fatigue-buffering effects of perceived social support may be more likely in high-intensity exercise contexts characterized by both positive sociality, support or camaraderie and high-stakes performance near the limits of exercise tolerance [33, 63, 78].

The results of this study should be considered in light of its limitations. First, the study was observational in nature and causality can only be inferred. Many variables potentially associated with sociality, affect or performance were not controlled for in the study design or analyses but could potentially affect outcomes. Nutritional intake and previous exercise in the period prior to the event might have affected performance, for example, though it is less obvious that these would systematically covary with the study’s social predictor variables. Higher feelings of energy could be predictive of higher sociability and perceptions of social support generally, though we are not aware of any evidence directly supporting this conjecture. More plausibly, higher performance outputs could prompt reports of higher subjective energy, via positive feedback mechanisms. Although some parkrunners track their times on personal watches, not all participants would have been aware of their objective performance during or immediately after the event. Surveys were sent out 45 min after the beginning of each parkrun. The median time taken to return the survey was approximately 4.5 hours, and 20% of surveys were returned over 24 hours after they were received (see S1 SOM of S3a Fig). As all returned surveys were included in analyses, it is likely that some participants would have accessed their 5 km run times before completing the survey. Post-hoc appraisals of subjective energy could therefore have been informed by knowledge of objective run time data. However, analyses show no effect of response time on variables related to participants’ affective experiences (i.e., their self-reports of fatigue, energy, or enjoyment) or their scores on the parkrun community component (see S1 SOM of 5 and S2–S5 Tables), and there is no theoretical reason to assume that this prior knowledge would affect reports of pre-run sociality or who participants reported coming or meeting up with at parkrun.

Previous qualitative research also offers some support for the hypothesized causality. Participants’ statements indicate awareness and appreciation of social support received from other parkrunners, including boosting effects on performance [42, 43]: “When people come through the finish line, you know, there’s people there and they’re all cheering you on and… it just gives a real boost” [64; p. 10]. The link to adherence is also explicit in these reflections: “Running with others is a massive motivation. … I don’t think I would run 5 km every week if I didn’t have a group like this to run with” [43; p. 12]. Further research is required to examine this link further, and to assess whether post-event affective responses (as measured here) influence or are otherwise related to pre-event and in-task responses. In this study, measures were not assessed during the event to minimize intrusiveness and influence on participants’ experiences [46].

Second, although our primary statistical analyses accounted for the repeated measures design, multilevel model results are influenced most by those participants with relatively high numbers of survey responses [79]. However, there are few significant differences between participants with high survey response counts (nine or more responses) and those with low survey response counts (fewer than nine responses; see S1 SOM of 5 and S1 Table). Nevertheless, future studies should strive for larger samples and for a higher median survey response number, where possible, to assess the replicability of results.

Third, the study does not investigate factors predicting variation in perceptions of social support, or in the association between social predictors and positive experiences or performance outcomes. It is important to note that the presence of others, even family and friends, does not always serve as a cue of social reward or social support. Furthermore, being the recipient of support is not always a positive experience. For example, highly neurotic individuals might tend to focus on the interpersonal costs of receiving social support while highly independent individuals can react to social support with feelings of compromised independence [80]. These responses have been theorized to reduce (or even abolish) the positive effects of social support on coping with stressors [80, 81]. Future research could examine effects of relationship quality and personality on experiences in exercise, as well as the effects of different exercise contexts (e.g., primarily competitive or primarily cooperative) on the quality of participants’ social connections and relationships and on subjective energy and self-efficacy levels. This could inform a broader understanding of how these variables relate to exercise experiences and adherence [82–84]. Interventions could better leverage and target the potential of social connection, reward and support to benefit the exercise experiences and health outcomes of individuals across a wider range of personality traits and socio-cultural backgrounds [85].

Finally, this study was limited to just six neighboring parkrun venues. Testing the hypotheses across a larger number of venues and in geographically and culturally diverse settings, could offer greater confidence in the generalizability of results.

## Conclusions

Affective dimensions of physical exercise and social integration and belonging are fundamental to addressing global public health challenges of physical inactivity and social isolation or loneliness. Traditionally, physical (in)activity research and social psychological research on these issues have proceeded in parallel, occupying distinct academic domains. Despite promising recent examples [38, 60, 62, 86], cross-fertilization between domains is historically limited by parochial emphases on the social factors that influence either health and wellbeing or performance in physical activity and exercise. Whereas social-environmental approaches to physical inactivity have focused primarily on social-ecological conditions that promote or inhibit health-beneficial exercise behavior, social psychological approaches to exercise adherence and performance have focused overwhelmingly on social facilitation effects engendered via stress (at worst) or distraction (at best), or on the benefits of cohesion for adherence, performance and health in team or group settings with clearly defined boundaries.

Here, we suggest that these issues do not belong in any single discipline, and that approaches need to draw from traditionally disparate areas of science (e.g., sports science, evolutionary anthropology, social psychology) to effectively inform public policy and civic engagement [85]. The research here takes a broad interdisciplinary, evolutionary and psychological approach to human social behavior, drawing from an extensive literature demonstrating that humans derive intrinsic pleasure from connecting, coordinating and cooperating together, and that our cooperative sociality profoundly influences homeostatic function, wellbeing and health [87]. The findings motivate new orientations on old questions about the social determinants of engagement and performance in exercise, offer novel directions for research into the public health value of community-led sports and exercise initiatives, and contribute to our nascent understanding of the synergistic interdependencies between social, psychological and biological factors in homeostatic self-regulation in exercise.

## Supporting information

S1 SOM Supplementary online information. (DOCX)
