# Supplementary material for: Social reward and support effects on exercise experiences and performance: Evidence from parkrun
Source: PLoS One. 2021 Sep 15;16(9):e0256546. doi: 10.1371/journal.pone.0256546 (PMC8443045; doi:10.1371/journal.pone.0256546)
Supplement: S1 SOM — (DOCX) [file pone.0256546.s001.docx]

**Supplementary Online Material**

**Social reward and support effects on exercise experiences and performance: evidence from parkrun**

Arran J. Davis^1^, Pádraig MacCarron^2^, Emma Cohen^1,3*^

^1^ Social Body Lab, Institute of Human Sciences, University of Oxford, 64 Banbury Road, Oxford OX2 6PN, UK

^2^ Mathematics Applications Consortium for Science and Industry, University of Limerick, Limerick, Ireland. V94 T9PX

^3^ Wadham College, Parks Road, Oxford OX1 3PN, UK

AD ORCiD: <https://orcid.org/0000-0002-8561-7768>

PM ORCiD: <https://orcid.org/0000-0002-5163-9264>

EC ORCiD: <https://orcid.org/0000-0002-5465-3440>

*Corresponding author

E-mail: [emma.cohen@anthro.ox.ac.uk](mailto:emma.cohen@anthro.ox.ac.uk) (EC)

1. **Research context of parkrun**

The aim of parkrun is to “promote physical activity and community spirit, by providing supportive opportunities to exercise” (1), p. 171). Organized by local volunteers and describing itself as a ‘run and not a race’ with no ability or attendance requirements, parkrun offers a welcoming, community-based setting for participants to attend as often as they please. This inclusive ethos allows parkrun to attract participants who do not identify with traditional views on competitive running, thus creating a participating population that includes a wide range of demographic categories and skill levels (1, 2). Researchers have described parkrun as a “mass community event” and a “home away from home” that offers participants “psychological comfort, warmth, and mutual support” (2; p. 10). While parkrun strives to create an inclusive ethos – it has a relatively even gender split (reported as a binary variable), and all age groups are well-represented across sites – it should be noted that individuals from ethnic minorities and low-socio-economic status (SES) groups are underrepresented at parkrun, including in areas that are ethnically diverse (3).

Parkrun offers a suitable naturalistic setting in which to study how social connectedness and support influence exercise outputs and experiences. According to a study conducted at one UK parkrun site, ‘social togetherness’ was the second most important aspect of parkrun among surveyed participants, following only “getting exercise” (2). Current literature on parkrun and our own ethnographic observations in the context of this research suggest that social relationships are built and solidified through interactions among parkrunners before and after runs (1, 2, 4). Individuals report chatting while running alongside other parkrunners, and often begin casual conversations as they wait in line (ordered by finishing rank) to scan their parkrun ID barcodes (used to record run times). Although participants at a given parkrun location can vary from week to week, there is a continuity of core runners and volunteers that work to create a sense of community (5), which offers runners an informal network of emotional support where “even loose ties with relative strangers can be highly valued” (2, p. 10).

1. **Survey recruitment and study dates**

Recruitment for the study ran from 23 January 2016 to 12 March 2016. The study lasted 18 weeks; the first surveys were sent out on 30 January 2016 and the last surveys were sent out on 28 May 2016.

1. **Additional data acquisition via parkrun websites**

A Python script was used to scrape publicly available data on 5 km run times and participant meta-data (age and sex) via parkrun websites. At the request of parkrun, this Python script is not shared publicly on the study [GitHub](https://github.com/arranjdavis/parkrun_survey_analyses_sociality_and_exercise_performance).

The script matched survey responses to runs in parkrun’s online database for 617 of 765 survey responses. The unresolved 148 responses were calculated manually (31 responses were duplicates or provided an unmatchable name and parkrun ID number, and the remaining 117 were from parkrunners who ran at locations different from the recruitment locations). Duplicate responses – multiple responses from the same participant on the same day – were removed from the data set (in this case, the participant’s first full response was maintained in the data set), as were survey responses for which no corresponding 5 km run time could be found.

1. **Multilevel model selection**

Due to the nested nature of the data (survey and run data within participants) we used multilevel linear regression models with participants as the level-two variable, allowing maximum random effects structures when possible (6).

Studies have shown biases in slope and intercept variance estimates when the number of level-one units (here: survey responses and 5 km run data) per level-two unit (here: participants) is small (7). However, it has been shown that this does not affect fixed effects estimates: “the proportion of singletons (up to .70) had no notable effect on the estimation of fixed effects for the level-one predictors, but evidenced a clear impact on the interval estimation of the parameters for the level-two predictors” (7; p. 4,060). Further, this study found that that:

with smaller levels of level-two units (less than 500), increases in the proportion of singletons led to a reduction in the accuracy of the confidence intervals for level-two predictors and bias in Type I error control of the binary level-two predictor, but did not impact the accuracy of the estimates for level-one predictors (7; p. 4,063).

Thus, estimates for the predictor variables of interest (all at level-one) should not have been affected by the large proportion of singletons in the data set.

In all cases, multilevel models were first fit using maximal random effects structures. When models failed to converge, random effects structures were simplified by eliminating random effects for covariates (smallest random effects variance first), followed by the social predictor variable, if necessary, until convergence was achieved (6).

1. **Correcting for family-wise error rates**

There are many ways to control for increased Type I error rates associated with making multiple comparisons. Particularly relevant to this study are methods that have the power to detect true effects while appropriately controlling the ‘false discovery rate’ (FDR) when hypothesis tests are dependent (8-10). As summarized in Table 1 of the main text, this study has six separate families of hypotheses.

The analyses reported in the main text used the following procedures, which call for the *p*-values in a family of tests to be ranked in ascending order (e.g., 1. *p* <.001, 2. *p* = .045, etc.) and then compared to a critical value calculated as:

(i / m) × Q

where i is the ordered rank of the *p*-value, m is the total number of tests in the family, and Q is the desired false discovery rate (9). In this study, m is always three (since each family consists of three hypothesis tests) and Q is always a (conservative) rate of 0.05. Thus, the critical values for each family of hypotheses are .017, .033, and .050 for the first, second, and third ranked *p*-values, respectively. Finally, critical values are compared to their corresponding *p*-values, starting with the lowest ranked (largest) *p*-value; all *p*-values lower than their critical values are accepted as significant, as are all *p*-values with ranks higher than the first *p*-value accepted as significant, even if they do not meet their respective adjusted critical value (9).

While this procedure is relatively straightforward for the families of comparisons compromising Hypothesis 1.1 – Hypothesis 4.6, it is less clear how it applies to mediation models, which test three separate, but related, pathways; indirect effects, direct effects, and total effects (11). Little research has been done on correcting for multiple comparisons when conducting (multilevel) mediation analyses (10). Given the lack of an established procedure, we treat each type of effect (indirect, direct, and total) as a sub-family of tests when accounting for multiple comparisons for Hypothesis 5.1 – Hypothesis 5.3 and Hypothesis 6.1 – Hypothesis 6.3, as the procedures used here are essentially testing whether the social predictor variables have significant indirect, direct, and/or total effects on 5 km run times. This will lead to, for example, the three indirect (or direct or total) effects generated from the tests of Hypothesis 5.1 – Hypothesis 5.3 and Hypothesis 6.1 – Hypothesis 6.3 being subjected to the procedures described in the previous paragraph.

1. **Principal components analysis (PCA) for parkrun community component**

A PCA was run on the two survey questions related to the parkrun community: “How much did you feel supported by the parkrun community today?” and “How much did you feel you were a part of the parkrun community today?” The two questions were highly correlated (*r* = .72).

Although an assumption of PCA is independence of data, which is violated by repeated measures designs, research has shown that PCA without accounting for serial correlation is acceptable (i.e., leads to negligible biases) when: (1) there are more than 30 level-two units (participants, in this case), (2) the correlations between the variables are high, and (3) researchers are only interested in the first principal component (12). This is the case in this instance, as there are 143 participants, the correlations between the variables are high (*r* = .72), and only one potential component was of interest.

Preliminary checks confirmed the usefulness of PCA in this context. Bartlett’s test was highly significant, indicating that PCA is appropriate for these variables, $\chi$^2^ (1) = 533.39, *p* < .001. The Kaiser-Meyer-Olkin (KMO) measure of sampling adequacy (MSA’s = 0.5, due to there only being two variables) suggested that the sample size and data were acceptable for PCA, and the determinant of the correlation matrix was unproblematic (0.482).

A single component was extracted from the two variables; it explained 86% of the variance in answers to the two questions and had good reliability (Cronbach’s alpha = .840). The parkrun community component scores were created by using the PCA coefficient scores from the analysis described above (*M* = 0, *SD* = 1).

- 1. **Check on whether using the parkrun community component biased model results**

Given the high correlation between the two variables that made up the parkrun community component (henceforth, constituent variables), all analyses involving the parkrun community component were also run using each of the two constituent variables, instead of the parkrun community component. This was done to test whether model results would have changed as a result of using a constituent variable instead of the parkrun community component.

Analyses revealed that using either of the constituent variables (i.e., responses to the questions about feeling ‘supported by the parkrun community’ and feeling ‘part of the parkrun community’) instead of the parkrun community component itself did not change model results.

That is, for all multilevel models that used the parkrun community component, statistical significance (at *p* = .05) was the same for the constituent variables as it was for the parkrun community component. This includes when the parkrun community component was used as a predictor (with subjective fatigue, subjective energy, subjective enjoyment, and 5 km run times as the outcome variable) and when it was the outcome (with logged response times as the predictor variable).

Results also remained the same for the multilevel mediation models. For the mediation analysis that tested whether participants’ perceived energy mediated the relationship between their scores on the parkrun community component and their (logged) 5 km run times, the significant average indirect effect of perceived energy remained statistically significant when using either of the constituent variables instead of the parkrun community component. For the mediation analysis that tested whether participants’ subjective fatigue mediated the relationship between their scores on the parkrun community component and their (logged) 5 km run times, the significant average indirect effect of subjective fatigue remained statistically non-significant when using either of the constituent variables instead of the parkrun community component.

Full results for all analyses reported in this section can be accessed by running this [script](https://github.com/arranjdavis/parkrun_survey_analyses_sociality_and_exercise_performance/blob/master/analysis_code/parkrun_survey_analysis.R) (see [here](https://github.com/arranjdavis/parkrun_survey_analyses_sociality_and_exercise_performance/blob/master/README.md) for instructions).

1. **Relationships between survey response count and outcome measures**

The survey response distribution was positively skewed (skewness = 0.986), such that 34 participants (22%) were responsible for 50% of all survey responses (*n* = 369). These 34 participants, henceforth ‘high responders’, had nine or more survey responses. ‘Low responders’ had fewer than nine survey responses, and these responses made up less than half of all returned surveys (*n* = 365). On average, high responders were older (*M* = 52.79 years, *SD* = 10.87 years) than low responders (*M* = 46.89 years, *SD* = 11.92 years), *t* (54.4) = 2.64, *p* = .011. The gender split between high (44% female) and low responders (50% female) was relatively even, $\chi^{2}$(1) = 0.14, *p* = .706.

Regarding the scores on the predictor, mediator, and outcome variables of interest, there were two significant differences between high and low responders: high responders had relatively lower levels of pre-run sociality and slower 5 km run times. Of all surveys returned by high responders, 59% (*n* = 217) reported being social before the run, whereas 67% (*n* = 254) of surveys returned by low responders reported being social before the run, $\chi^{2}$(1) = 5.09, *p* = .024. High responders tended to have slower 5 km run times (*M* = 28 min 26 s, *SD* = 6 min 10 s) than did low responders (*M* = 27 min 17 s, *SD* = 5 min 31 s), *t* (726.9) = 2.54, *p* = .011 (*t*-test run on logged 5 km run times to improve model fit). See SOM S1 Table for a full summary of variable comparisons by respondent type.

1. **Relationships between survey response times and outcome measures**

The median survey response time (from when the link was sent) was 4 hr 32 min. The distribution was highly positively skewed (skewness = 2.98), with a range of 4 min 17 s to 6 days 22 hrs and 31 min, and a mean of 15 hr 32 min (*SD* = 26 hr 38 min). Of the 734 survey responses, 195 (27%) were returned within 2 hrs, 585 (80%) within one day, 664 (90%) within two days, and 717 (97%) within four days (see SOM S3a Fig). The median survey completion time (measured from the time participants clicked on the link to the survey to the time they submitted their final answer) was 2 min 39 s (*M* = 14 min 41 s, *SD* = 150 min 40 s, range = 56 s – 43 hr 10 min); 87% of surveys were completed in under 5 min, and 96% were completed in under 10 min (see SOM S3b Fig). There were no a priori exclusions based on time taken to return or complete surveys.

Multilevel models were used to assess whether survey response times (time elapsed from receiving the survey link to submission) affected responses to affect-related questions. In these models, logged (to improve model fits) response times were the predictor, and subjective fatigue, perceived energy, subjective enjoyment, and the parkrun community component were the outcomes. No covariates were included in these models. Response times did not significantly predict any of these variables (see S2 – S5 Tables).

1. **Statistical model assumption checks**
   1. **Assumption check methods**

Model assumptions were checked according to previously published methods (13). The assumption checks for all models can be acquired by running the main analysis [script](https://github.com/arranjdavis/parkrun_survey_analyses_sociality_and_exercise_performance/blob/master/analysis_code/parkrun_survey_analysis.R), which will produce a folder for each model that will contain test statistics and plots for all assumption checks. For all models in the study, the assumptions were either met or broken in way that did not bias results.

Given the similarity between models used in the study, and uniformity of the justifications used for ignoring broken assumptions, we formally report only the assumption checks for the multilevel mediation analysis used to test Hypothesis 5.1. We chose the assumption checks for the analyses used in testing Hypothesis 5.1 because it included both multilevel model *and* mediation model assumption checks, and a continuous (i.e., more complex) social predictor variable. Thus, these assumption checks are representative of those used for all other analyses in this study. Again, all information needed for the assumption checks not reported here are available through running the main analysis script.

- - 1. **Level-one residual homoscedasticity**

Level-one homoscedasticity was assessed by observing between-group (i.e., parkrunner) differences in level-one residual variance, acquired through running separate OLS regressions on the data from the parkrunners included in the main model. The variability of posterior means estimated by the multilevel model should be similar to variability of coefficients estimated by the separate OLS regressions.

Well-specified models should also have constant (i.e., homoscedastic) level-one residual variance with residual dispersion measures that are normally distributed. This can be tested using a (squared) standardized residual dispersion measure (*d*) against a null hypothesis of homoscedastic level-one residual variance; *d* will have a Gaussian distribution when there is level-one homoscedasticity (14). In order to avoid bias, constant level-one residual variance was tested using only the data from relatively large level-two groups (13); in this case, that meant using data from participants with at least 10 survey responses.

- - 1. **Level-one residual normality**

Normal probability plots of standardized OLS residuals were used to check level-one residual normality (13).

- - 1. **Level-one linearity**

Plots of unstandardized ordinary least squares (OLS) residuals against level-one explanatory variables (covariates) were used to check the linearity of fixed effects (13).

- - 1. **Level-two residual homoscedasticity**

Standardized level-two residuals were plotted against ‘relevant level-two variables’ (covariates with random slopes) to check for homoscedasticity (13). Due to convergence failures, none of the models in this study had covariates with random slopes – this assumption is thus not tested here.

- - 1. **Level-two residual normality**

Normal probability plots of standardized level-two residuals were used to check for level-two residual normality (13).

- - 1. **Level-two linearity**

Unstandardised level-two residuals were plotted as a function of relevant level-two variables (covariates with random slopes) to check for linearity (13). Due to convergence failures, none of the models in this study had covariates with random slopes – this assumption is thus not tested here.

- - 1. **Sequential ignorability**

Multilevel mediation models have all the assumptions listed above, plus one additional assumption; the sequential ignorability assumption, which assumes that there are no unobserved confounding variables. This assumption was checked using previously published methods (15).

- 1. **Assumption checks for Hypothesis 5.1**

Hypothesis 5.1 was that parkrun community component scores would positively predict perceived energy levels, which would be negatively related to 5 km run times (i.e., higher scores on the parkrun community component would predict higher perceived energy levels, and higher perceived energy levels would predict *faster* 5 km run times). A multilevel mediation analyses showed a significant, ergogenic indirect effect of the parkrun community component on 5 km run times, via a positive effect on perceived energy levels.

Below, we report assumption checks for the constituent multilevel models used in the Hypothesis 5.1 mediation analysis – the model using the parkrun community component to predict perceived energy levels (model ‘a’), and the model using perceived energy levels to predict 5 km run times, while controlling for the parkrun community component (model ‘b’). We also report the assumption check for the mediation itself.

- - 1. **Constituent model ‘a’: the parkrun community component on perceived energy levels**
       1. **Level-one homoscedasticity**

S7 Fig shows that the variability of posterior means estimated by the multilevel model are similar to the variability of coefficients estimated by the separate OLS regressions, indicating level-one homoscedasticity (13). The only exceptions to this are extreme coefficient estimates resulting from small sample sizes (i.e., from participants with relatively few survey responses); for example, 83% of participants with outlying coefficient estimates (± 3 *SD*) for the parkrun community component had fewer total survey responses than the overall average of 5.13 responses per participant. This suggests that heteroscedastic coefficient estimates were due largely to participants with relatively few survey responses, which is common and unproblematic (13).

Nevertheless, the null hypothesis of level-one homoscedasticity was rejected for this data, *H* = 24.59, *df* = 14, *p* = .039, indicating level-one heteroscedasticity (as the standardized residual dispersion measure, *d*, was not Gaussian). However, the Q-Q plot of *d* reveals that it is close to being normally distributed (see S8a Fig). The value of *H* also depends on the normality of the level-one residuals; heavier-tailed distributions of level-one residuals will cause inflated values of *H*, even if the residuals have (relatively) constant variance (13). The level-one residuals for the current model are indeed heavily tailed (see S8b Fig). Given the heavy tail of level-one residuals, that heteroscedasticity was caused largely by participants with relatively few observations, and that *d* is relatively normally distributed, it can be assumed that the model has an unproblematic amount of heteroscedasticity (13).

- - - 1. **Level-one residual normality**

S8b Fig shows that the normality assumption has been violated, due to longer tails than what would be expected with the normal distribution; specifically, the residuals display positive skew. However, it has been demonstrated that fixed effects estimates are robust to violations of the normality assumption; multilevel models with non-normally distributed level-one residuals do not produced biased fixed effect parameter estimates (16).

- - - 1. **Level-one linearity**

Level-one linearity was confirmed by plots of the unstandardized OLS residuals against the only relevant level-one predictor – whether participants reported slowing down to run with a running partner (-1), running at a natural pace (0), or speeding up to run with a running partner (1) on their runs. Residuals were centered around 0 at all levels of this predictor (see S9 Fig).

- - - 1. **Level-two residual normality**

Normal Q-Q plots of standardized level-two residuals for random intercepts (due to convergence issues, this model had no random slopes) revealed slight deviations from normality for the random intercepts (see S10 Fig). However, it has been shown that estimates of fixed effects and their standard errors are robust to non-normal level-two residual errors when there are at least 50 level-two units (17, 18); this study had 143 level-two units (i.e., participants). Given this, it can be assumed that fixed effect estimates (and their standard errors) are unbiased.

- - - 1. **Influential level-two units**

Cook’s distances revealed that no participants had an undue influence on the model (*M* = 0.01, *SD* = 0.01, range = < .001 – 0.07). Further, the significance of the model results did not change with the removal of any of the level-two units.

- - 1. **Constituent model ‘b’: perceived energy on logged 5 km run times, while controlling for the parkrun community component**
       1. **Level-one homoscedasticity**

S11 Fig shows that the variability of posterior intercept means estimated by the multilevel model are similar to the variability of coefficients estimated by the separate OLS regressions, indicating level-one homoscedasticity (13). Posterior slopes are constant for this model – due to convergent failures, the model had no random slopes.

- - - 1. **Level-one residual normality**

S12a Fig shows that the normality assumption has been violated, due to longer tails than what would be expected with the normal distribution. However, it has been demonstrated that fixed effects estimates are robust to violations of the normality assumption; multilevel models with non-normally distributed level-one residuals do not produced biased fixed effect parameter estimates (16).

- - - 1. **Level-one linearity**

Level-one linearity was confirmed by plots of the unstandardized OLS residuals against the only relevant level-one predictor – participants’ perceived energy levels. Residuals were centered around 0 at all levels of this predictor (see S12b Fig).

- - - 1. **Level-two residual normality**

Normal Q-Q plots of standardized level-two residuals for random intercepts (due to convergence issues, this model had no random slopes) revealed slight deviations from normality for the random intercepts (see S13 Fig). However, it has been shown that estimates of fixed effects and their standard errors are robust to non-normal level-two residual errors when there are at least 50 level-two units (17, 18); this study had 143 level-two units (i.e., participants). Given this, it can be assumed that fixed effect estimates (and their standard errors) are unbiased.

- - - 1. **Influential level-two units**

Cook’s distances revealed that no level-two units (i.e., participants) had an undue influence on the model (*M* = 0.02, *SD* = 0.09, range = < .001 – 0.73). Further, the significance of the model results did not change with the removal of any of the level-two units.

- - - 1. **Sequential ignorability**

Regarding the additional assumption for mediation analyses (here, unobserved confounders), tests revealed that the model met the sequential ignorability assumption (see S14 Fig) *if* the product for the coefficients for (potential) unobserved confounders and the mediator and outcome are positive, as indirect effects are estimated to be negative in this scenario, which is in accordance with the findings of the mediation analysis being checked here (13, 15, 19).

The model is less robust to potential confounders with a negative relationship to both the mediator and outcome, as all indirect effect estimates are positive in this scenario (contrary to the effect observed in the mediation analysis being checked here). However, it is not obvious what such a confounder would be, as this variable would need to correlate negatively with perceived energy and 5 km run times.

**Figures**


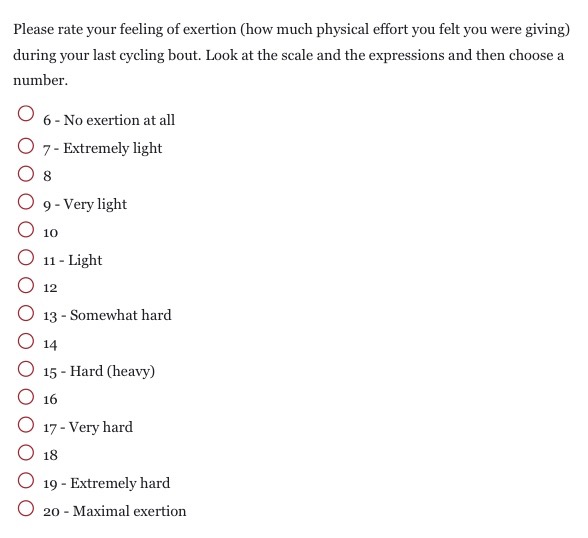


**S1 Fig.** Borg Scale of Perceived Exertion. This scale is prefaced with: “Please rate your feeling of exertion (how much physical effort you felt you were giving) during your run today.”


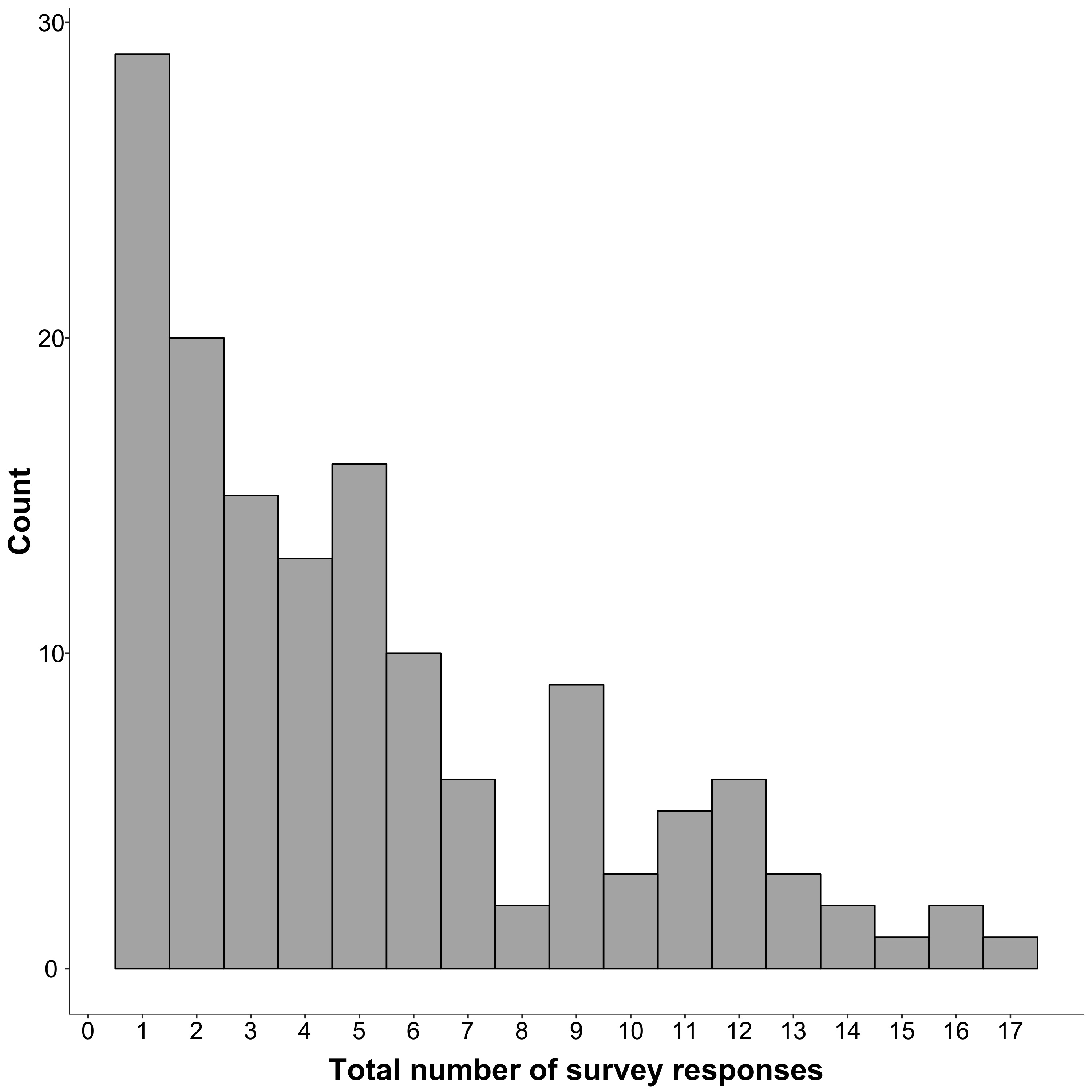


**S2 Fig.** Histogram of participants’ total survey responses.

| **(a)**  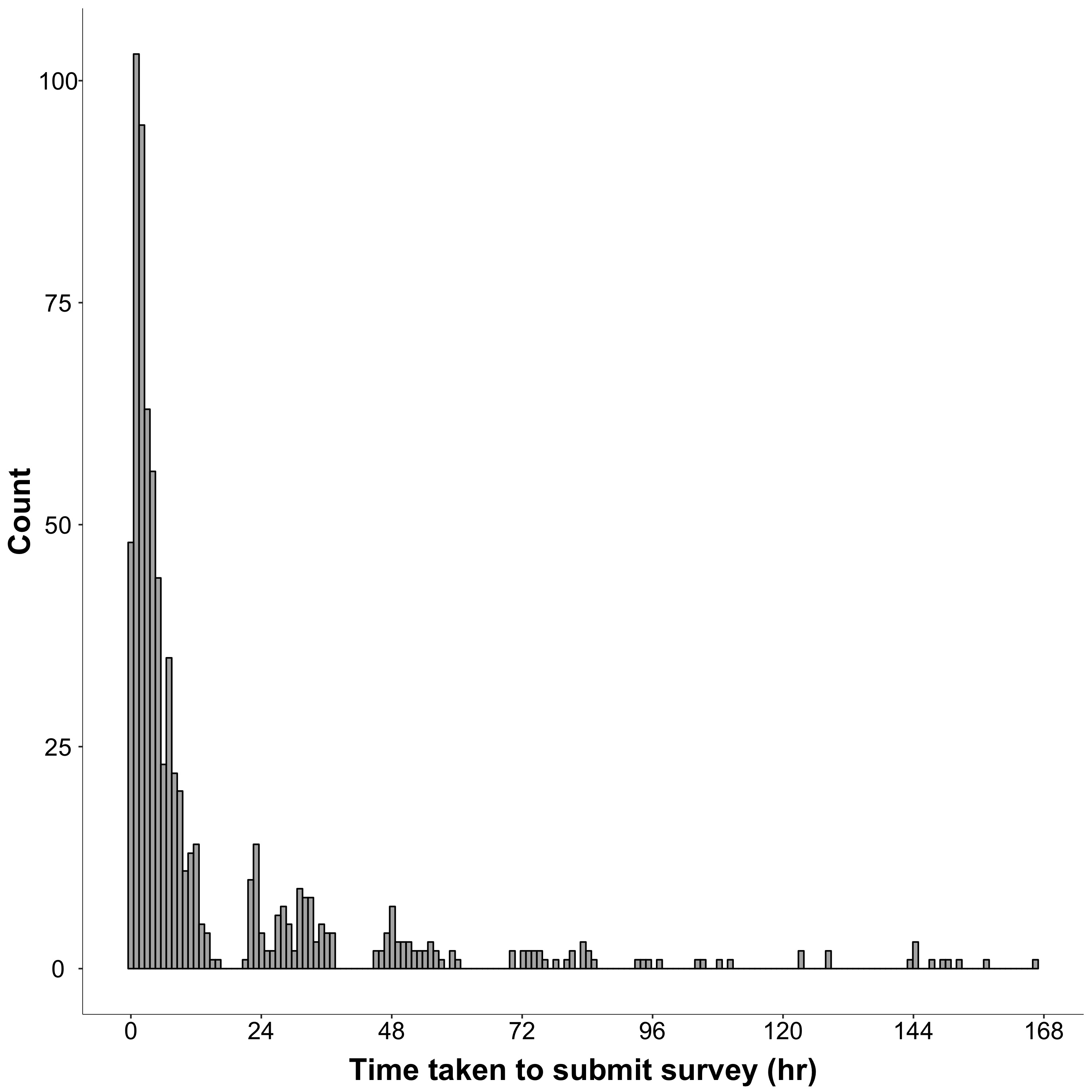 | **(b)**  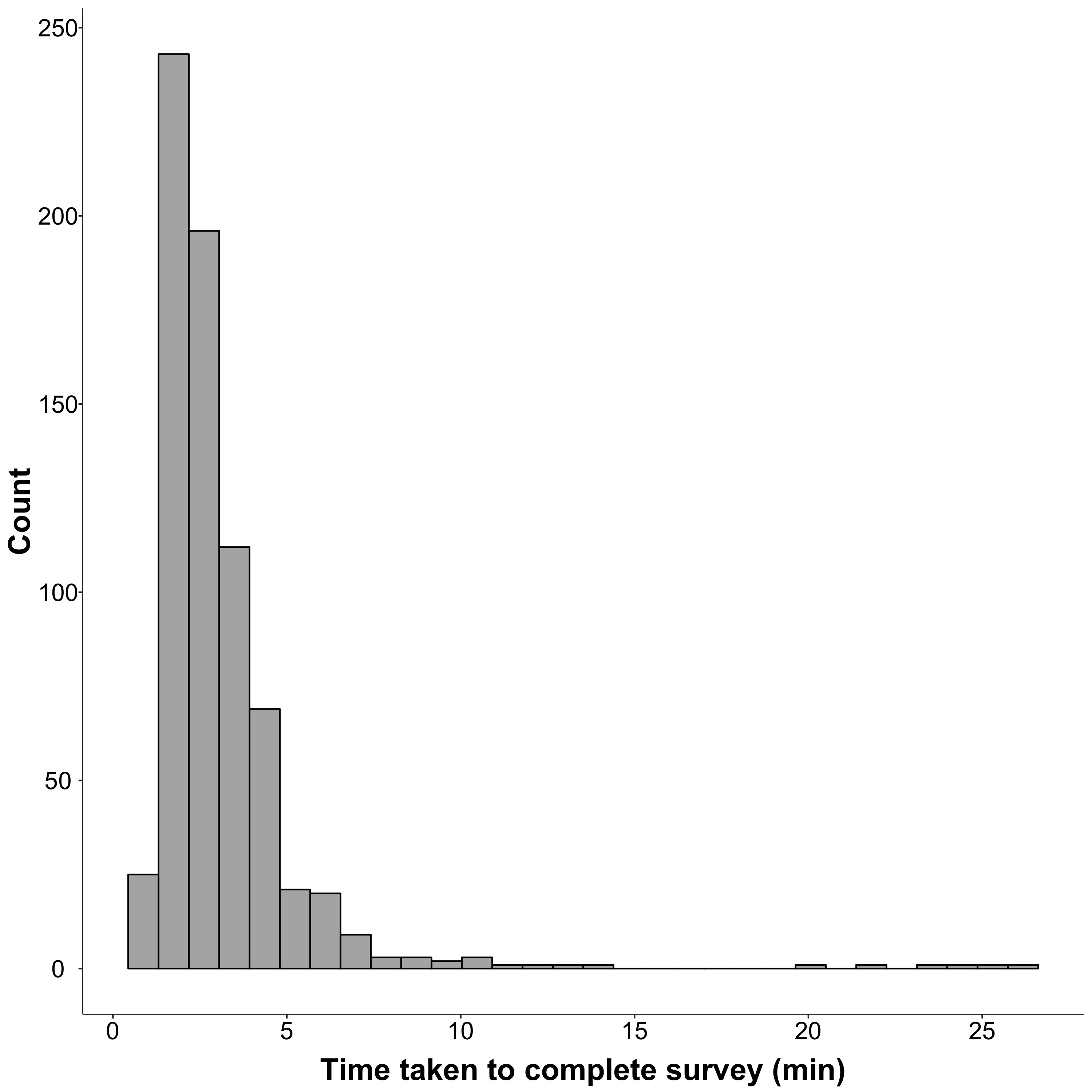 |
| --- | --- |
| **S3 Fig.** Histograms of (a) time taken (from the nearest Saturday at 9:45 a.m.) to return completed surveys and (b) time taken to complete the survey (measured from the time participants clicked on the link to the survey to the time they submitted their final answer); all surveys that took longer than 30 min were excluded from the histogram to facilitate its interpretation. Of all completed surveys included in analyses, 3% took longer than 30 min to complete. This is likely due to one of two reasons, both relating to the fact that start times were derived from when participants first clicked on the link to the survey. Participants with unusually long completion times may have either clicked the link, but then delayed beginning the survey, or completed the survey, but then delayed submitting their final answer. | |


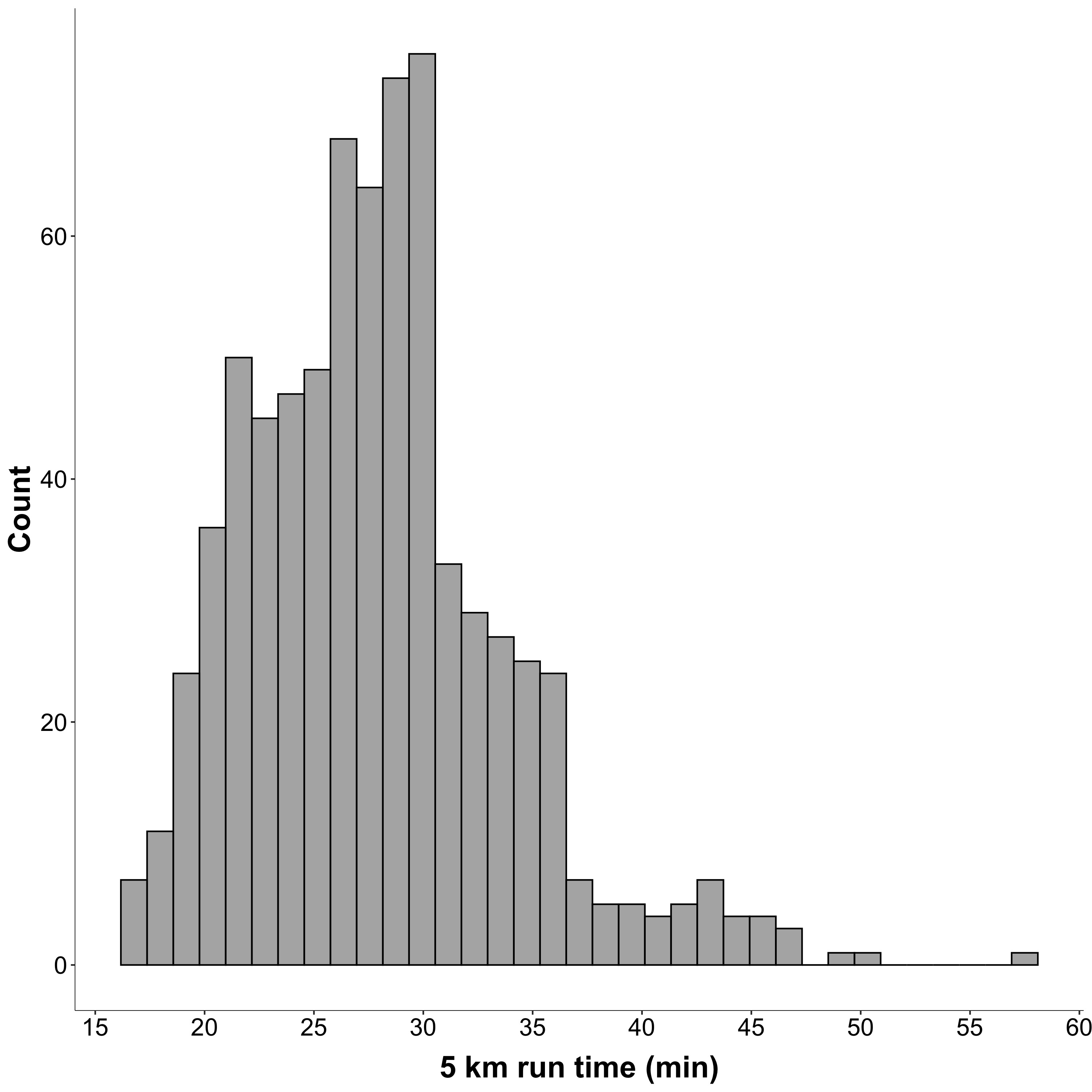


**S4 Fig.** Histogram of 5 km run times associated with participant responses.

| **(a)**  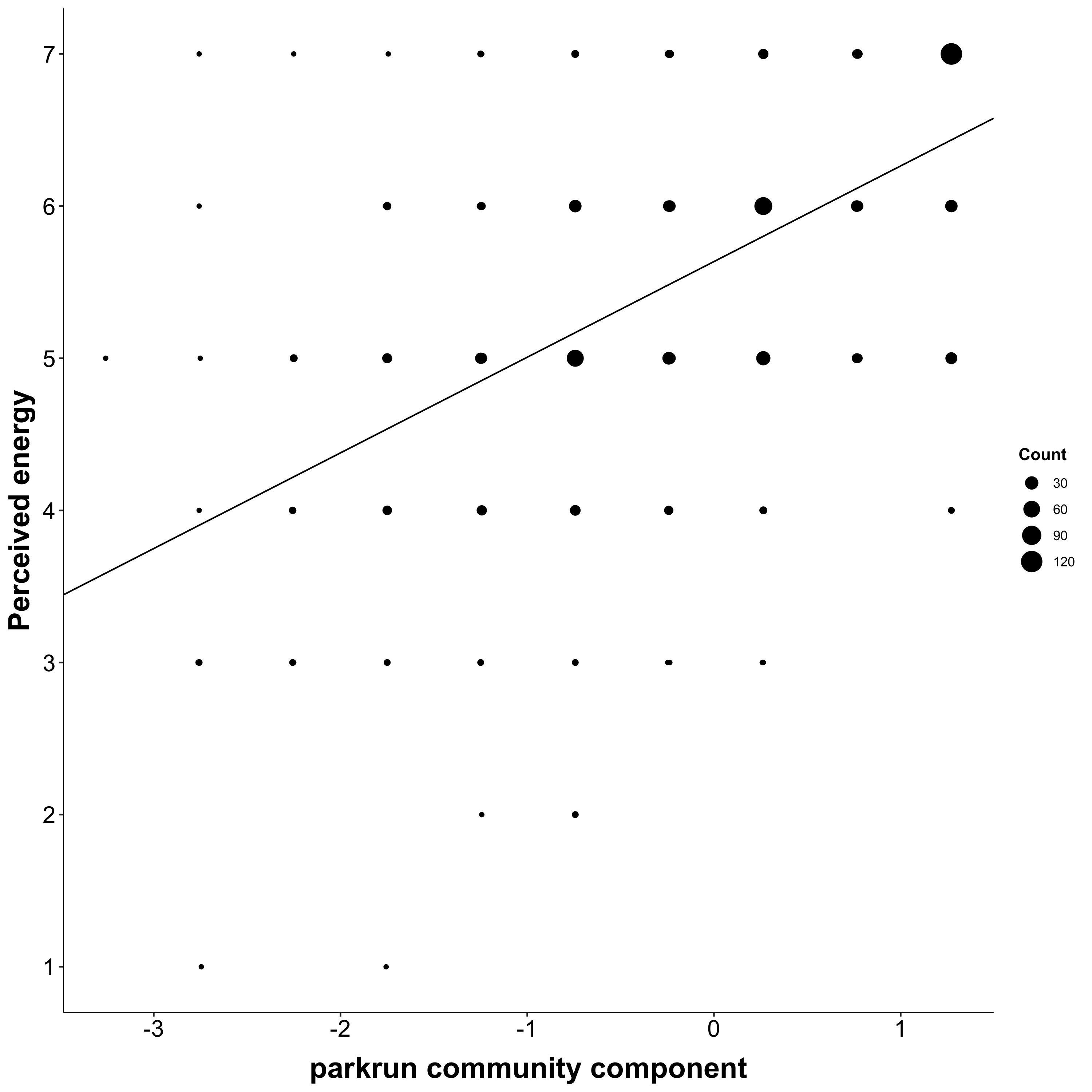 | **(b)**  **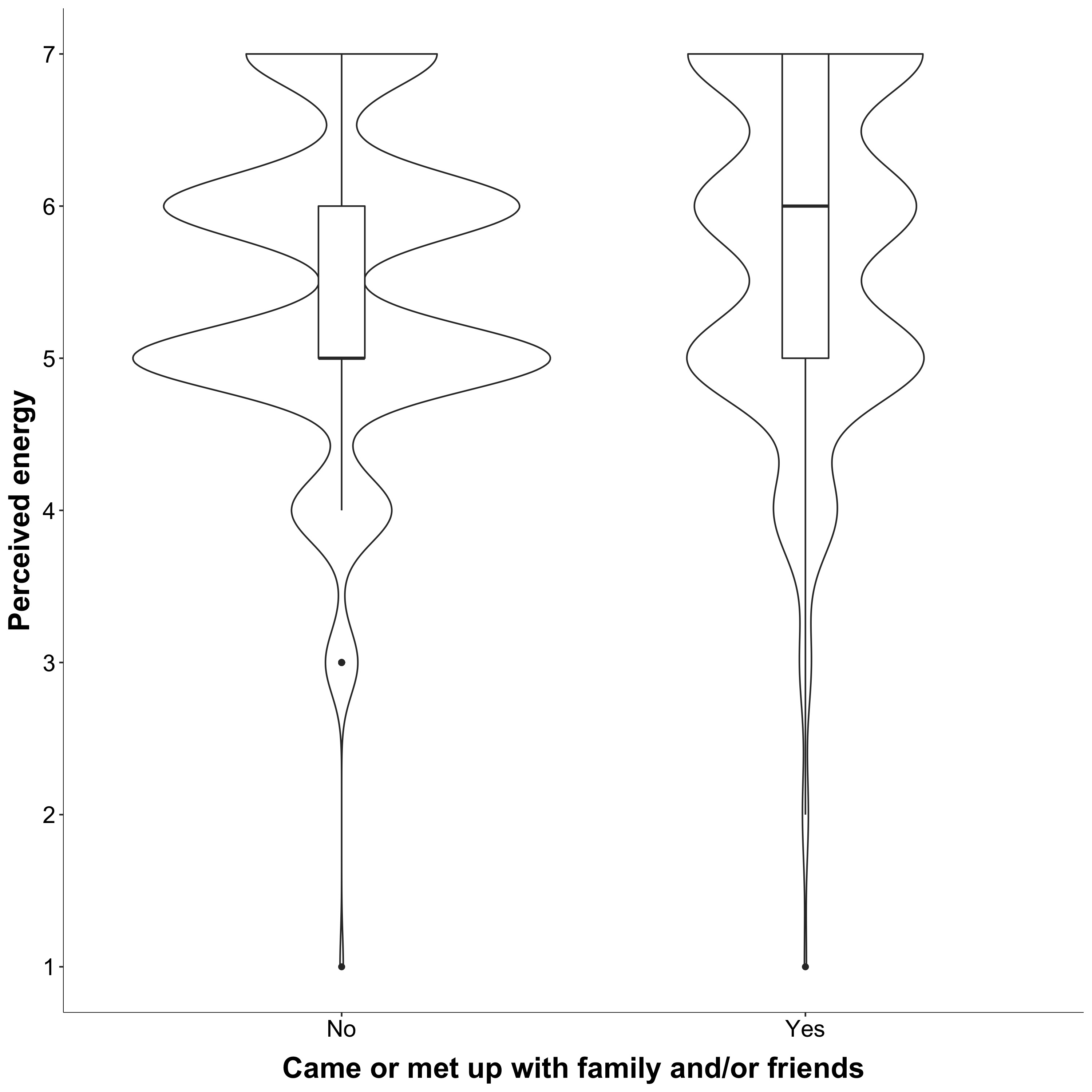** |
| --- | --- |
| **(c)**  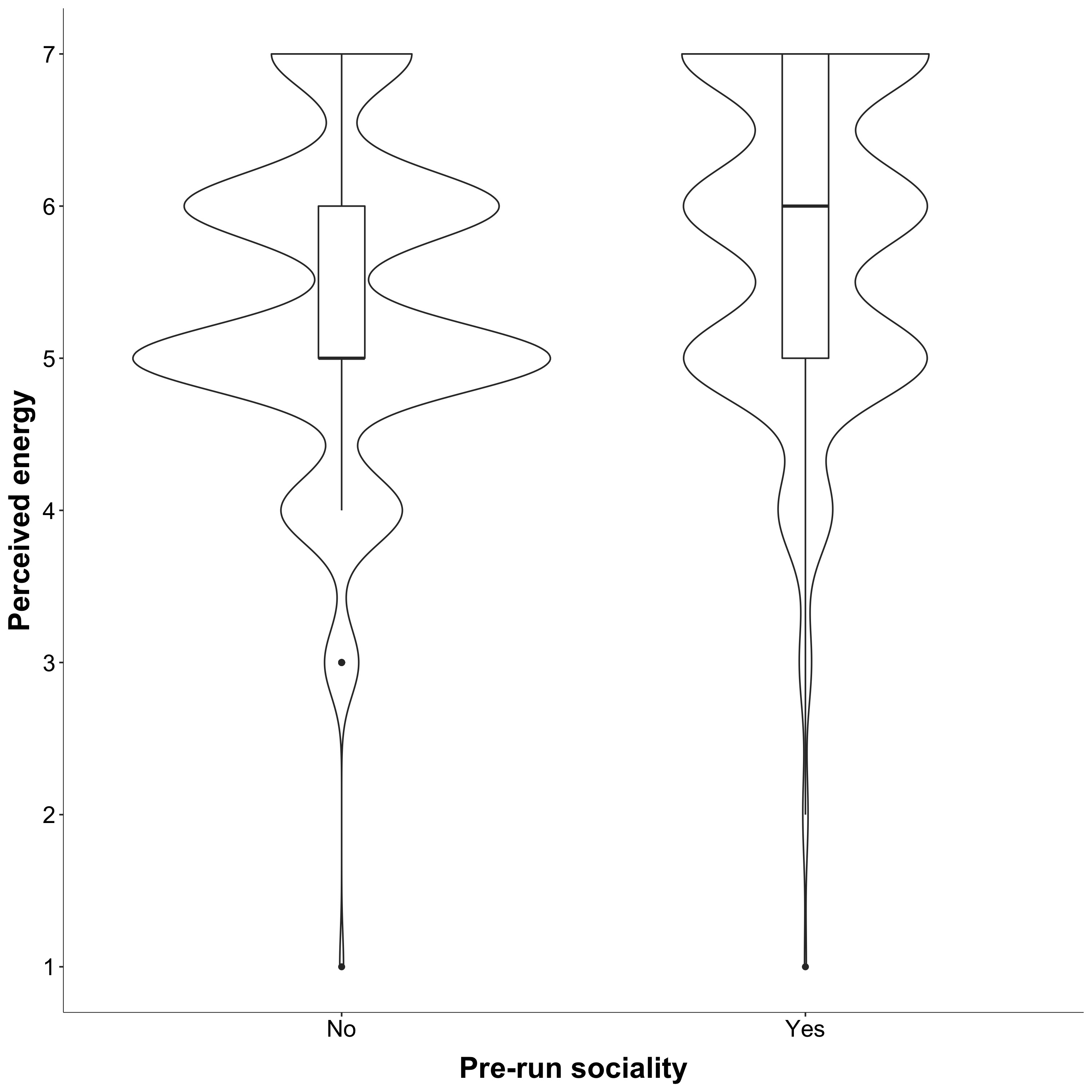 | **(d)**  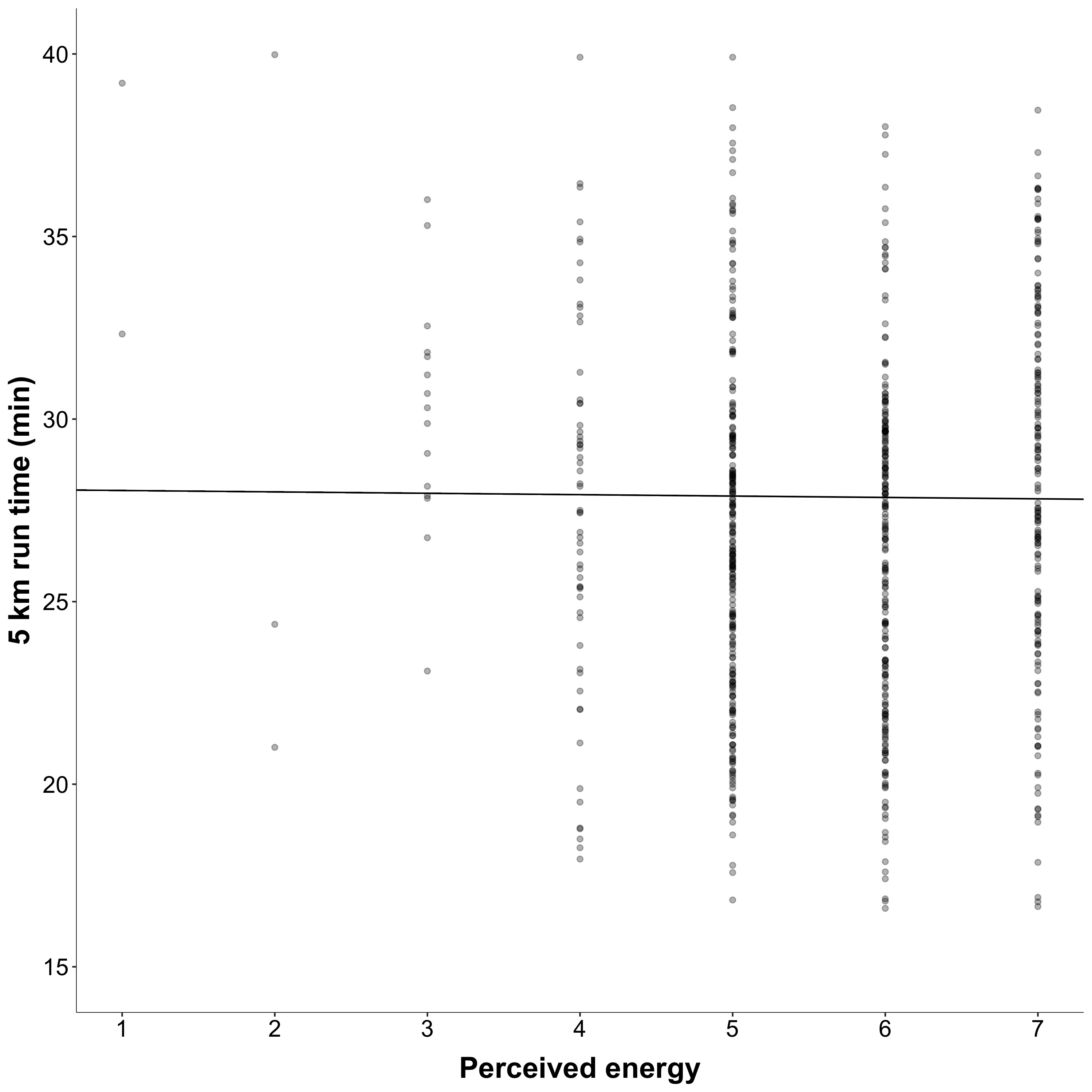 |
| S5 Fig. (a) Scatter plot of the relationship between participants’ parkrun community component score and their levels of perceived energy (regression line produced by regressing perceived energy scores on parkrun community component scores), (b) violin plot (with box plot underlay) of the relationship between whether or not participants came or met up with family and/or friends at parkrun and their levels of perceived energy, (c) violin plot (with box plot underlay) of the relationship between participants’ pre-run sociality and their levels of perceived energy, and (d) scatter plot of the relationship between participants’ levels of perceived energy and their 5 km run times (in order to facilitate interpretation, runs greater than two standard deviations from the mean – here, those lasting longer than 40 min – are not shown; regression line produced by regressing all unlogged 5 km run times on perceived energy scores). | |

|  |
| --- |
| **S6 Fig.** Mediation diagram depicting the direct, indirect, and total effects of the social predictor variables – the parkrun community component (Prediction 6.1), whether or not participants came or met up with family and/or friends (Prediction 6.2), their score on the score on), and their pre-run sociality (Prediction 6.3) – on 5 km run times, with participants' subjective fatigue as a potential mediator. |

| 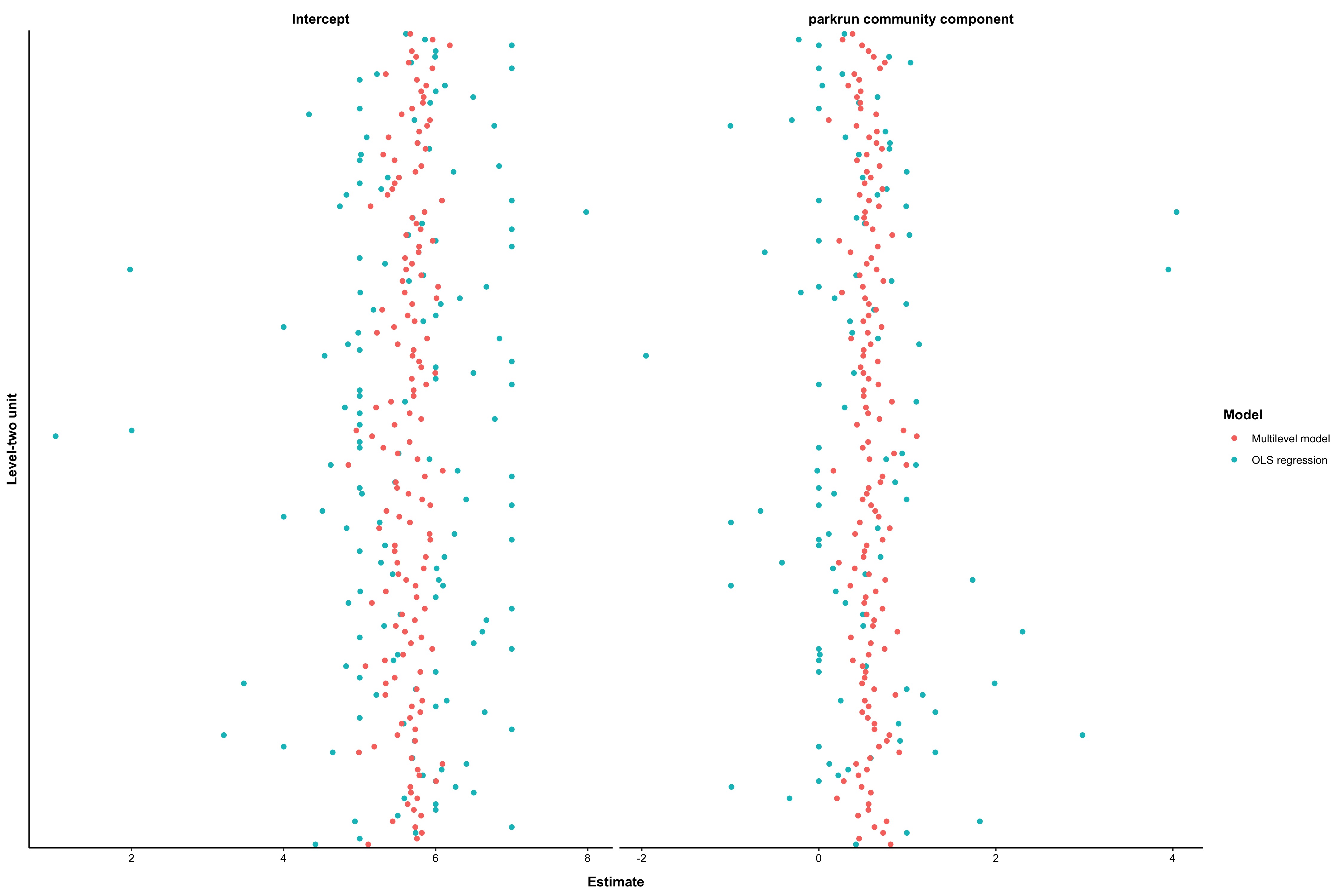 |
| --- |
| **S7 Fig.** OLS coefficient estimates for each participant (blue) versus the posterior means for each participant in the multilevel model (pink). Both models used the parkrun community component as the social predictor variable, and perceived energy levels as the outcome. One extreme outlier was removed from the plot to facilitate observation, this participant was included in all other analyses. |

| **(a)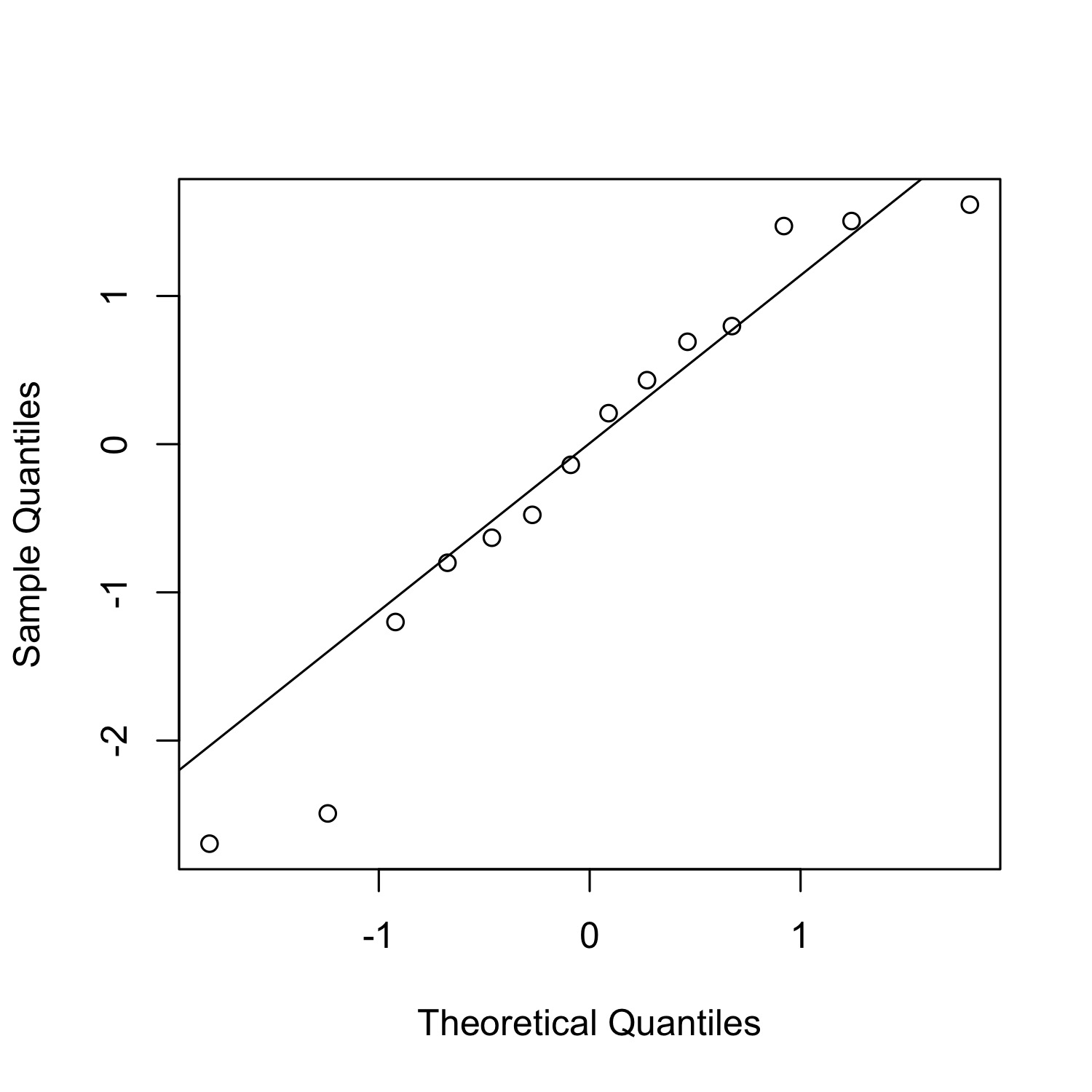** | **(b)**  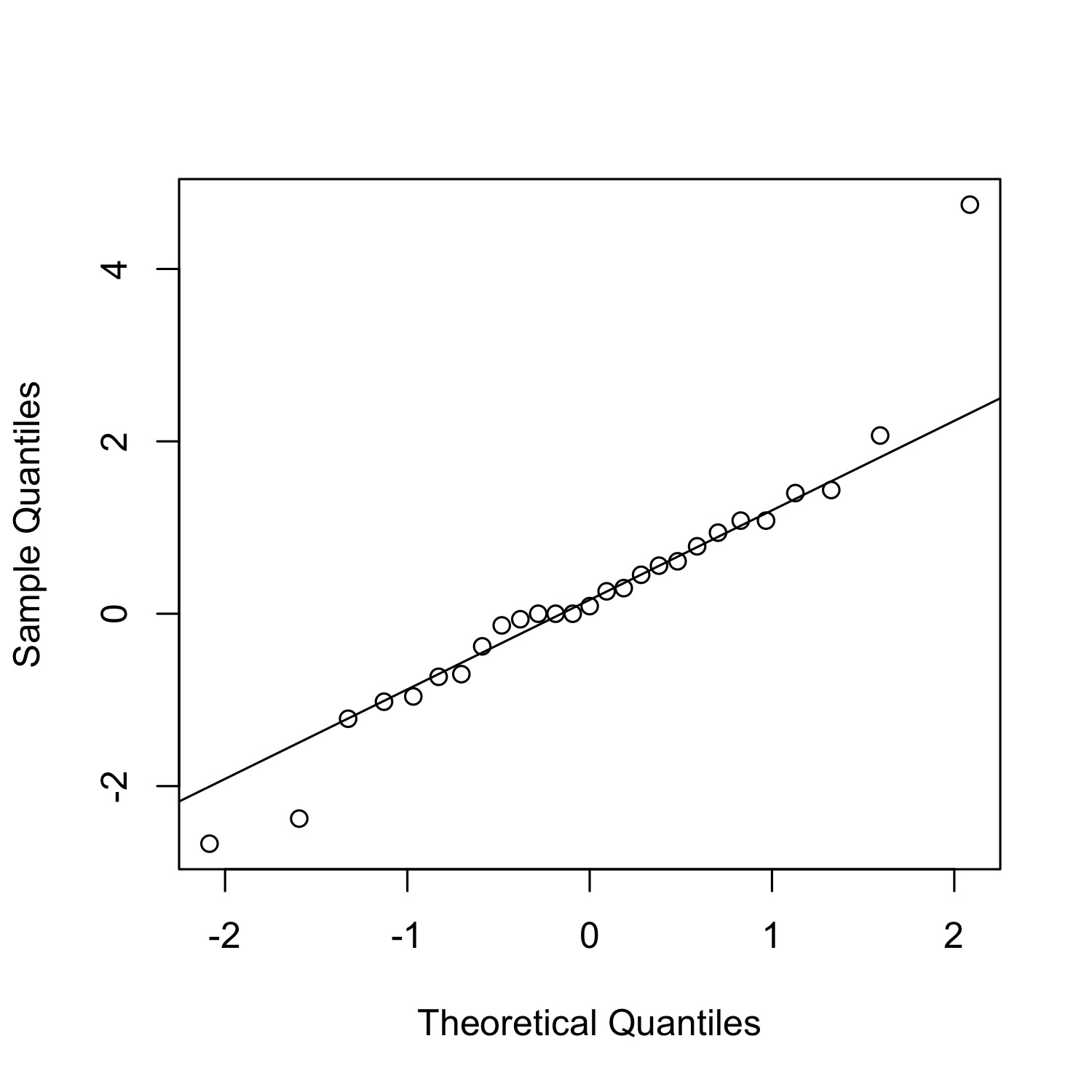 |  |
| --- | --- | --- |
| **S8 Fig.** (a) Q-Q plot of the standardized residual dispersion measure, *d* and (b) level-one residuals for multilevel model on perceived energy levels. | | |

| 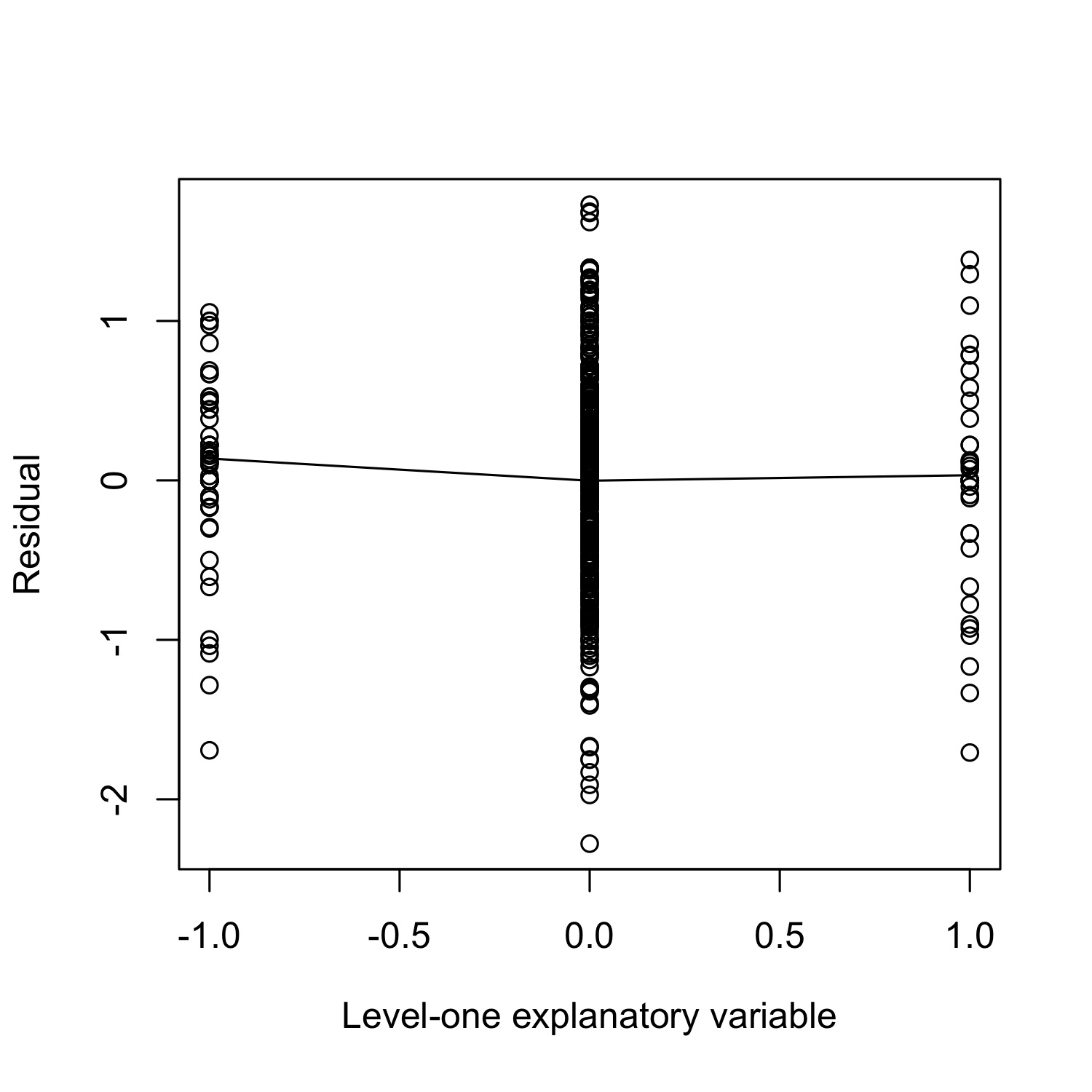 |
| --- |
| **S9 Fig.** Unstandardised OLS residuals with LOWESS (locally weighted scatterplot smoothing) line for the only level-one covariate: whether participants reported slowing down to run with a running partner (-1), running at a natural pace (0), or speeding up to run with a running partner (1) on their runs. |

| 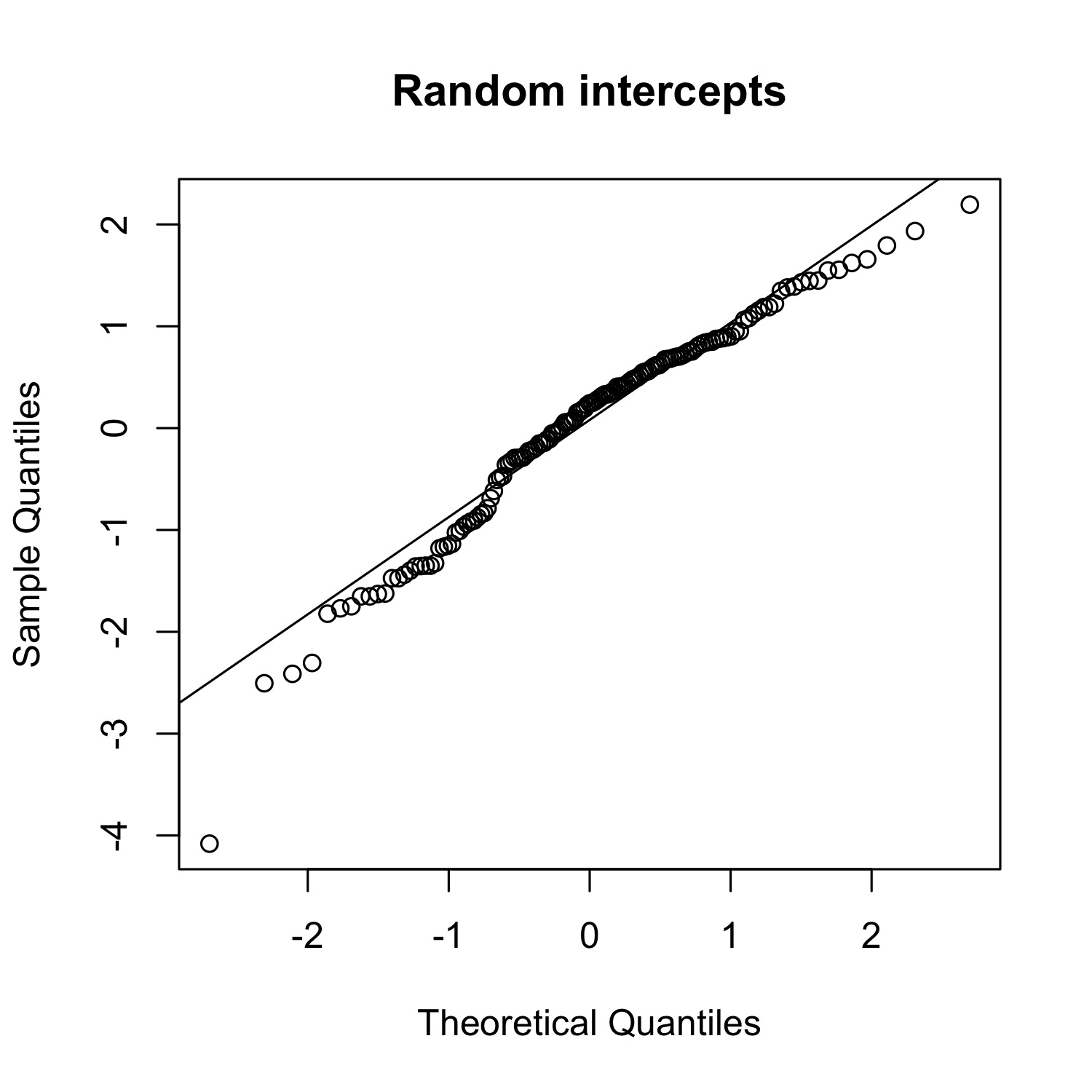 |
| --- |
| **S10 Fig.** Standardized level-two intercept residuals versus the expected order statistics of a normal distribution (Q-Q plot). |

| 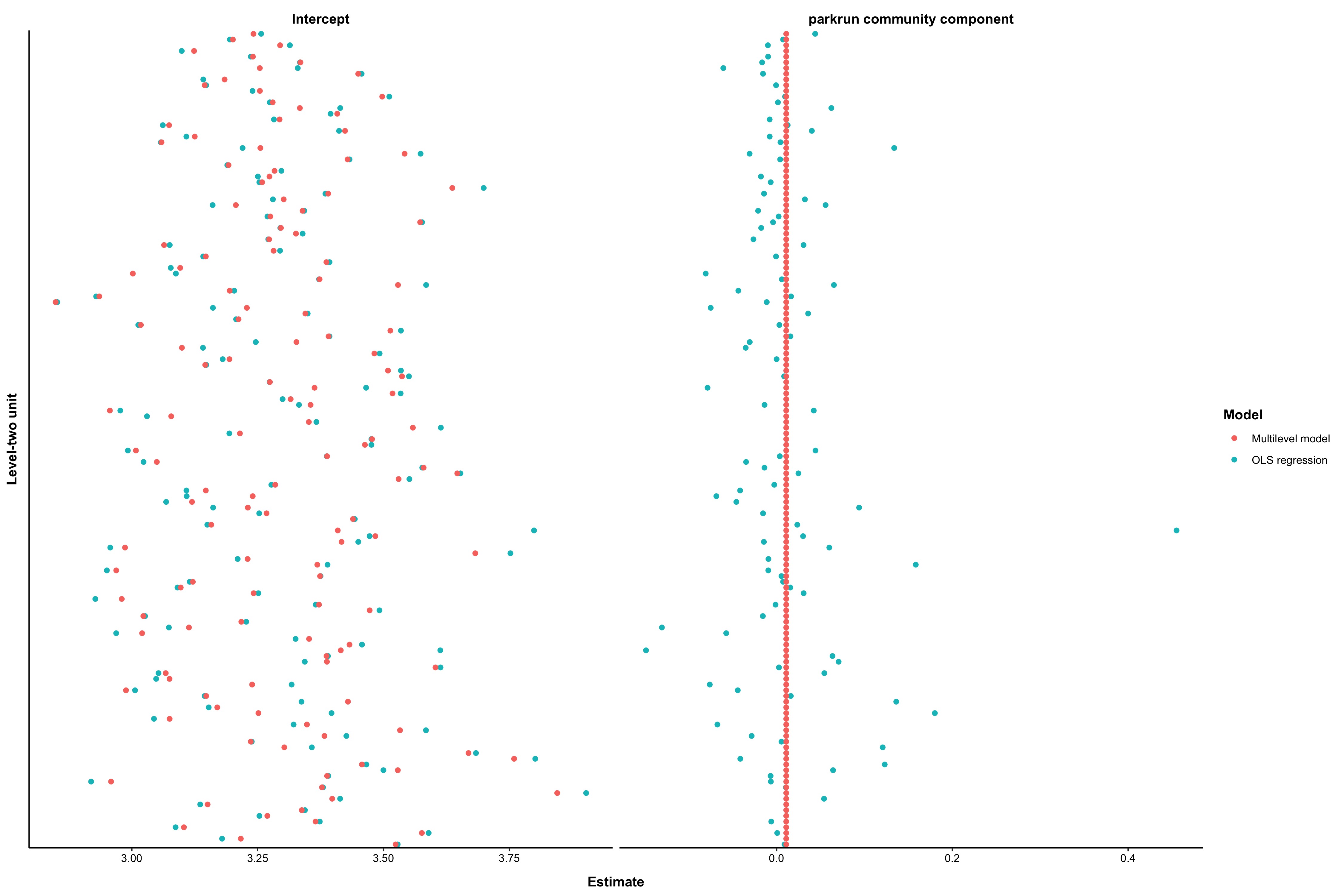 |
| --- |
| **S11 Fig.** OLS coefficient estimates for each participant (blue) versus the posterior means for each participant in the multilevel model (pink). Both models used the parkrun community component as the social predictor variable, and perceived energy levels as the outcome. One extreme outlier was removed from the plot to facilitate observation, this participant was included in all other analyses. |

| **(a)**  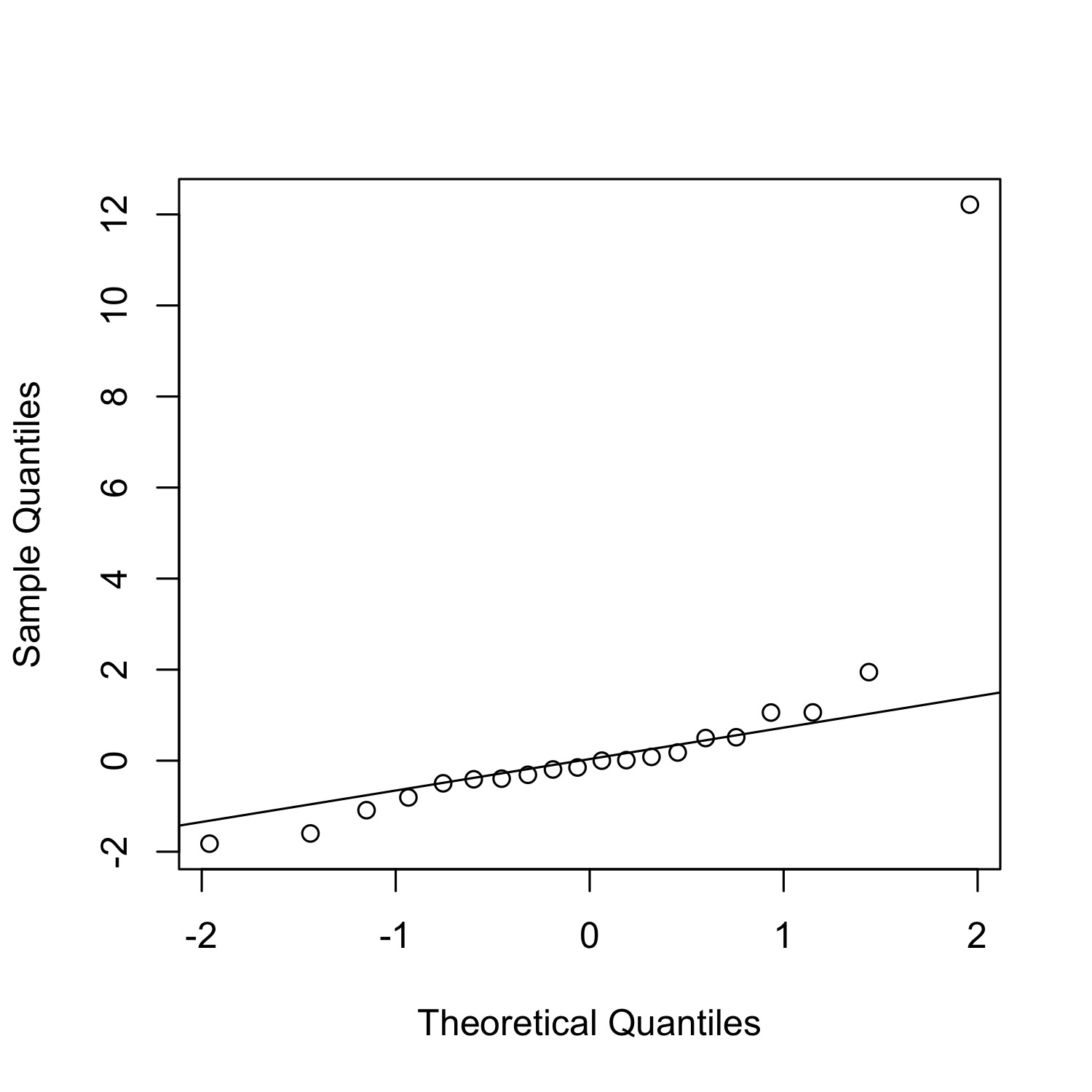 | **(b)**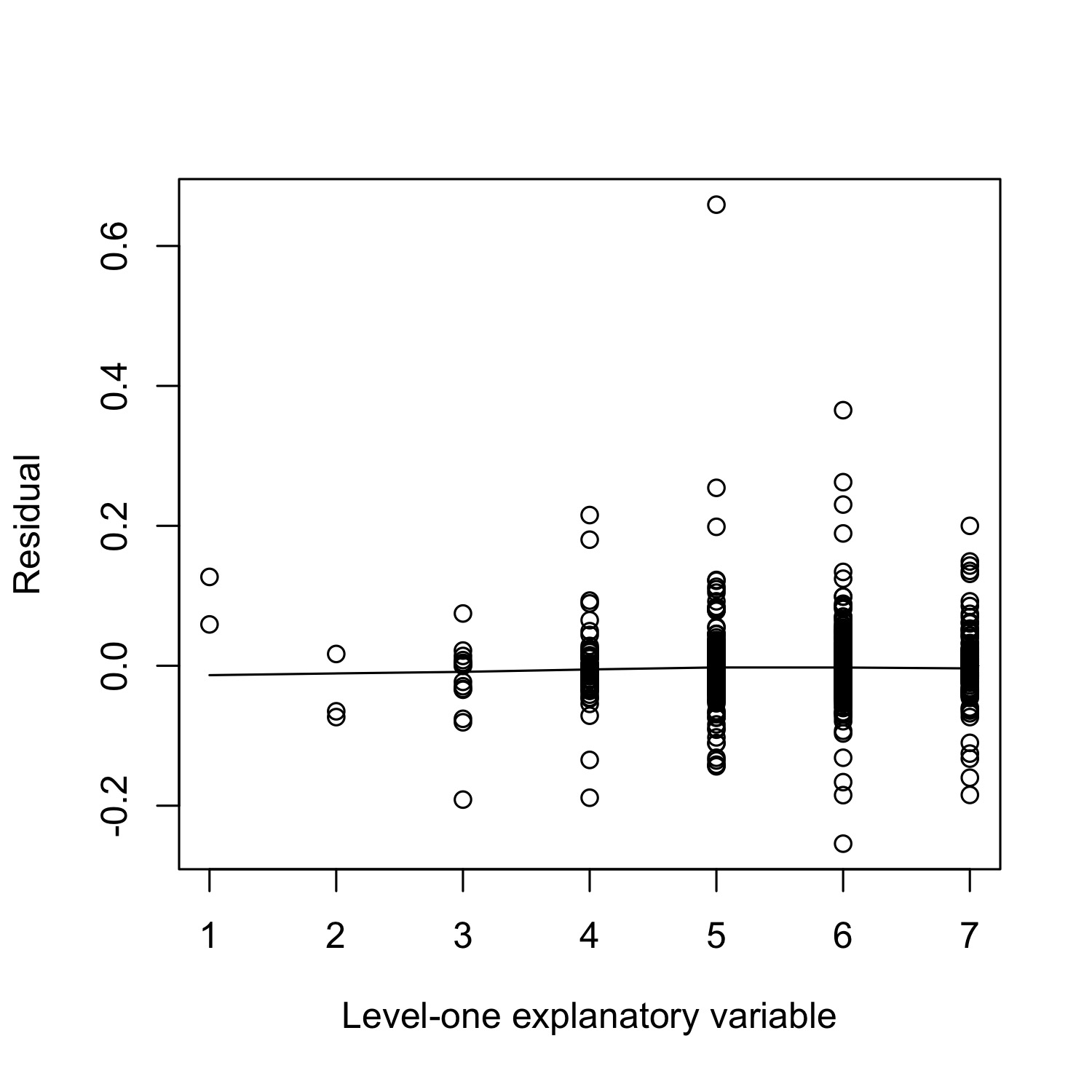 |
| --- | --- |
| **S12 Fig.** (a) Q-Q plot of level-one residuals for multilevel model on perceived energy levels and (b) unstandardized OLS residuals with LOWESS (locally weighted scatterplot smoothing) line for the (only) level-one explanatory variable: perceived energy levels. | |

| 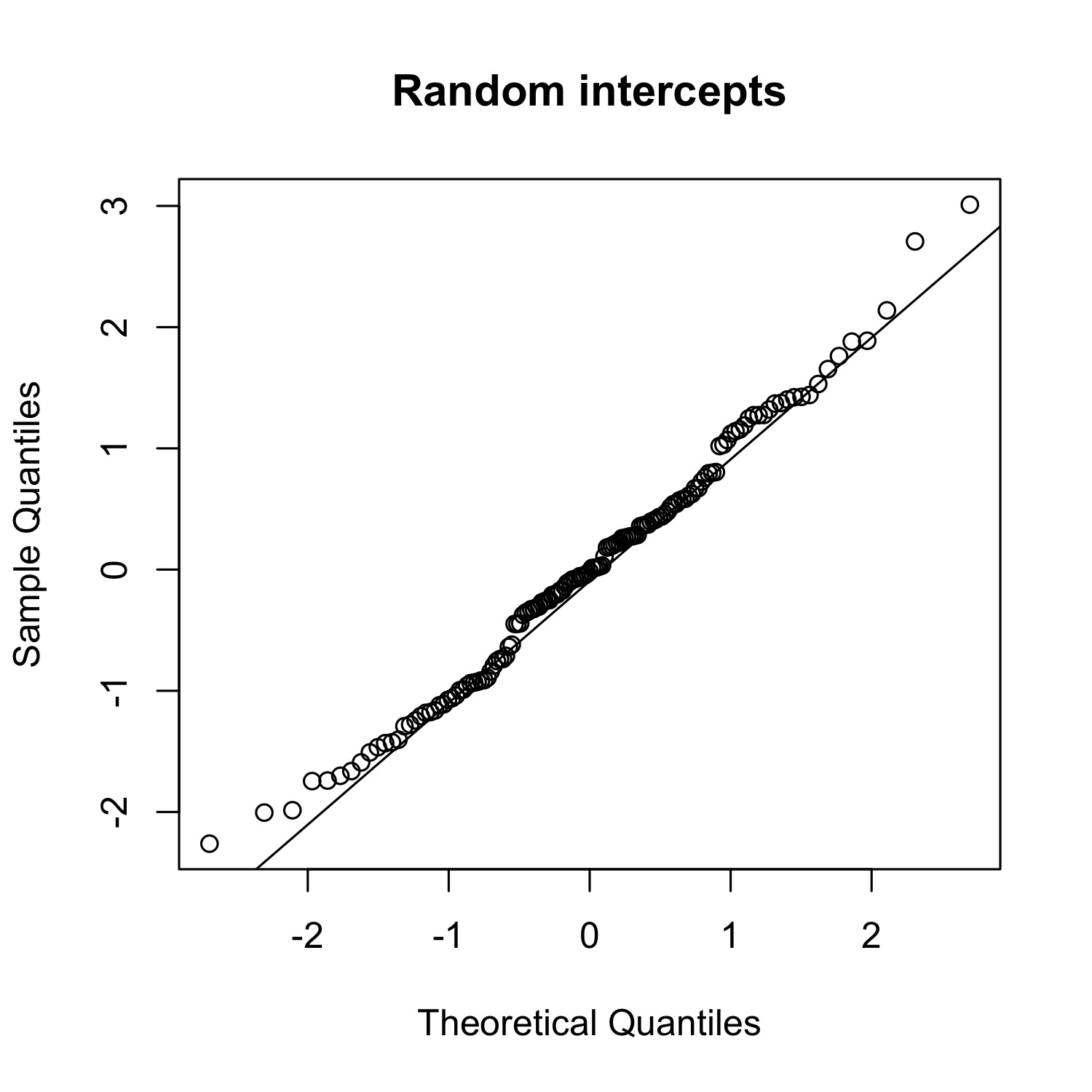 |
| --- |
| **S13 Fig.** Standardized level-two intercept residuals versus the expected order statistics of a normal distribution (Q-Q plot). |

| 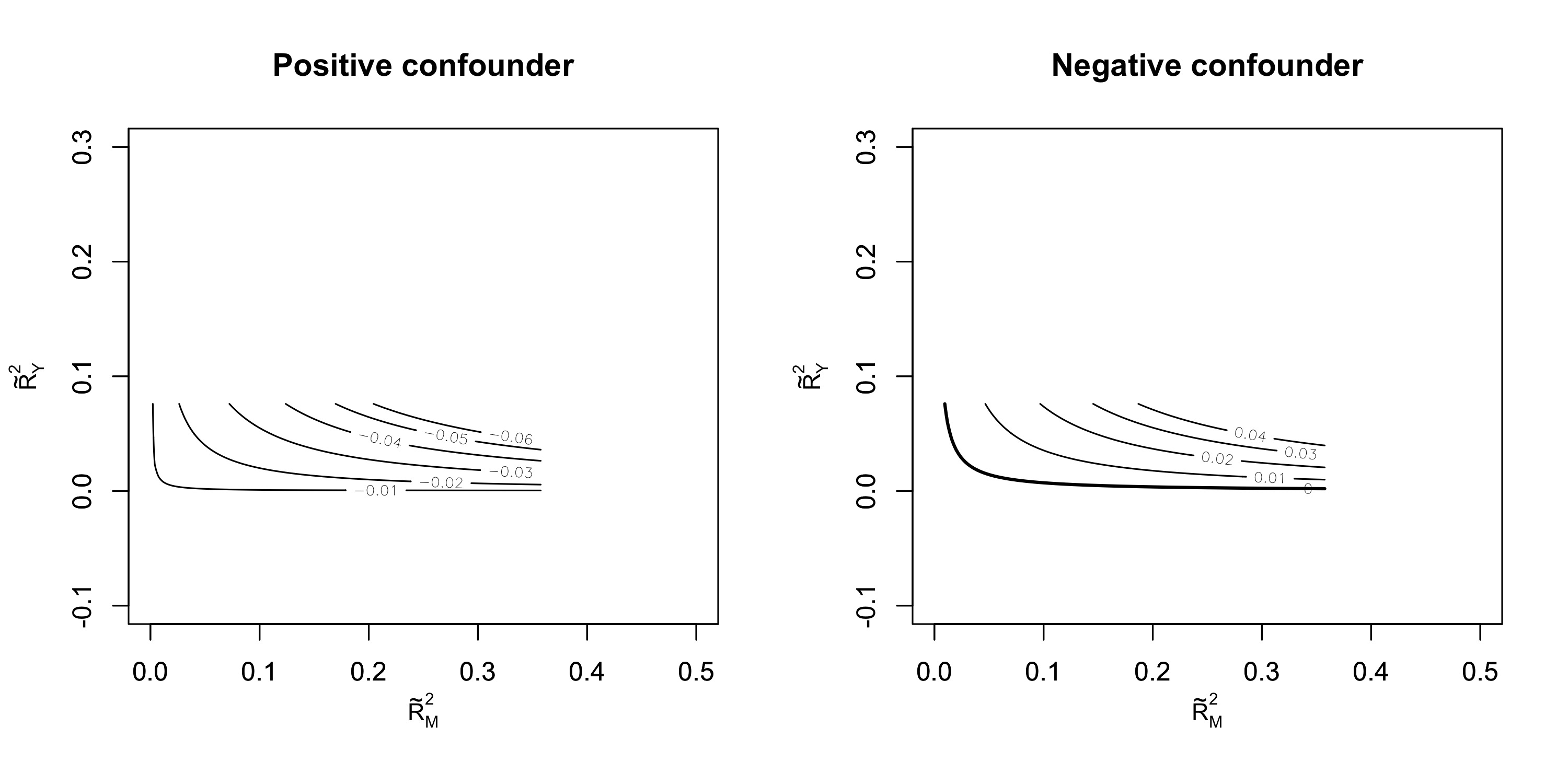 |
| --- |
| **S14 Fig.** Sequential ignorability assumption check for positive and negative confounders. |

**Tables**

| **S1 Table.** Comparisons of predictor, mediator, and outcome variable scores by participant response count (high v. low) | | | | |
| --- | --- | --- | --- | --- |
| **Variable** | **Response count** | **Measure** | **Test statistic (*df)*** | ***p*** |
| Community component | High | −0.014 (0.950) ^a^ | −0.394 (723.2) ^d^ | .694 |
|  | Low | 0.015 (1.049) ^a^ |  |  |
| Came or met up with family and/or friends | High | 48.78% ^b^ | 1.611 (1) ^e^ | .204 |
|  | Low | 43.36% ^b^ |  |  |
| Pre-run sociality | High | 58.81% ^c^ | 5.089 (1) ^e^ | .024 |
|  | Low | 67.12% ^c^ |  |  |
| Perceived energy | High | 5.564 (1.046) ^a^ | −1.854 (729.4) ^d^ | .064 |
|  | Low | 5.709 (1.072) ^a^ |  |  |
| Fatigue | High | 4.821 (1.266) ^a^ | 0.574 (731.6) ^d^ | .566 |
|  | Low | 4.767 (1.283) ^a^ |  |  |
| 5 km run times | High | 28 min 26 s (6 min 10 s) ^a^ | 2.537 (726.9) ^d^ | .011 |
|  | Low | 27 min 17 s (5 min 31 s) ^a^ |  |  |
| Participants classified as having high response counts had nine or more total survey responses, whereas those classified as having low response counts had fewer than nine total survey responses.  ^a^ *M* (*SD*)  ^b^ Percentage of surveys where participants reported coming/meeting up with friends/family  ^c^ Percentage of surveys where participants reported being social before the run  ^d^ Two-tailed *t*-test  ^e^ Chi-square test for equality of proportions | | | | |

| **S2 Table.** Effect of survey response time on subjective fatigue | | | | |
| --- | --- | --- | --- | --- |
| **Variable** | ***b*** | ***SE*** | ***t* (*df*)** | ***p*** |
| Intercept | 4.912 | 0.193 | 25.44 (268.4) | < .001 |
| Logged response time | −0.024 | 0.032 | −0.76 (612.6) | .449 |
| **Random part** | **Variance** | ***SD*** |  |  |
| Intercept | 0.253 | 0.503 |  |  |
| Logged response time | < 0.001 | 0.015 |  |  |
| $\text{R}_{\text{m}}^{\text{2}}$ = < 0.001, $\text{R}_{\text{c}}^{\text{2}}$ = 0.209 | | | | |

| **S3 Table.** Effect of survey response time on perceived energy | | | | |  |
| --- | --- | --- | --- | --- | --- |
| **Variable** | ***b*** | ***SE*** | ***t* (*df*)** | ***p*** | |
| Intercept | 5.919 | 0.163 | 36.42 (67.5) | < .001 | |
| Logged response time | −0.048 | 0.025 | −1.92 (68.1) | .060 | |
| **Random part** | **Variance** | ***SD*** |  |  | |
| Intercept | 0.566 | 0.752 |  |  | |
| Logged response time | 0.002 | 0.047 |  |  | |
| $\text{R}_{\text{m}}^{\text{2}}$ = 0.005, $\text{R}_{\text{c}}^{\text{2}}$ = 0.397 | | | | | |

| **S4 Table.** Effect of survey response time on subjective enjoyment | | | | |
| --- | --- | --- | --- | --- |
| **Variable** | ***b*** | ***SE*** | ***t* (*df*)** | ***p*** |
| Intercept | 5.906 | 0.166 | 35.44 (245.2) | < .001 |
| Logged response time | −0.035 | 0.027 | −1.32 (730.8) | .186 |
| **Random part** | **Variance** | ***SD*** |  |  |
| Intercept | 0.359 | 0.599 |  |  |
| Logged response time | < 0.001 | 0.002 |  |  |
| $\text{R}_{\text{m}}^{\text{2}}$ = 0.003, $\text{R}_{\text{c}}^{\text{2}}$ = 0.302 | | | | |

| **S5 Table.** Effect of survey response time on parkrun community component | | | | |
| --- | --- | --- | --- | --- |
| **Variable** | ***b*** | ***SE*** | ***t* (*df*)** | ***p*** |
| Intercept | −0.006 | 0.153 | −0.04 (68.9) | .968 |
| Log 5 km run time | −0.007 | 0.024 | −0.28 (59.5) | .781 |
| **Random part** | **Variance** | ***SD*** |  |  |
| Intercept | 0.593 | 0.770 |  |  |
| Log 5 km run time | 0.005 | 0.073 |  |  |
| $\text{R}_{\text{m}}^{\text{2}}$ < 0.001, $\text{R}_{\text{c}}^{\text{2}}$ = 0.488 | | | | |

| **S6 Table.** Effect of parkrun community component on perceptions of fatigue | | | | |  |
| --- | --- | --- | --- | --- | --- |
| **Variable** | ***b*** | ***SE*** | ***t* (*df*)** | ***p*** | |
| Intercept | 4.786 | 0.068 | 70.67 (105.1) | < .001 | |
| Community component | 0.066 | 0.061 | 1.08 (71.2) | .284 | |
| **Random part** | **Variance** | ***SD*** |  |  | |
| Intercept | 0.269 | 0.519 |  |  | |
| Community component | 0.129 | 0.359 |  |  | |
| $\text{R}_{\text{m}}^{\text{2}}$ = 0.002, $\text{R}_{\text{c}}^{\text{2}}$ = 0.244 |  |  |  |  | |

| **S7 Table.** Effect of pre-run sociality on perceptions of fatigue | | | | |  |
| --- | --- | --- | --- | --- | --- |
| **Variable** | ***b*** | ***SE*** | ***t* (*df*)** | ***p*** | |
| Intercept | 4.809 | 0.097 | 49.83 (258.7) | < .001 | |
| Pre-run sociality | −0.054 | 0.104 | −0.519 (702.7) | .604 | |
| **Random part** | **Variance** | ***SD*** |  |  | |
| Intercept | 0.346 | 0.588 |  |  | |
| Pre-run sociality | 1.308 | 1.144 |  |  | |
| $\text{R}_{\text{m}}^{\text{2}}$ < 0.001, $\text{R}_{\text{c}}^{\text{2}}$ = 0.209 |  |  |  |  | |

| **S8 Table.** Effect of who participants came or met up with on perceptions of fatigue | | | | |  |
| --- | --- | --- | --- | --- | --- |
| **Variable** | ***b*** | ***SE*** | ***t* (*df*)** | ***p*** | |
| Intercept | 4.889 | 0.075 | 64.46 (83.8) | < .001 | |
| Came or met up with family and/or friends | −0.250 | 0.111 | −2.245 (92.7) | .027 | |
| **Random part** | **Variance** | ***SD*** |  |  | |
| Intercept | 0.155 | 0.394 |  |  | |
| Came or met up with family and/or friends | 0.317 | 0.563 |  |  | |
| $\text{R}_{\text{m}}^{\text{2}}$ = 0.009, $\text{R}_{\text{c}}^{\text{2}}$ = 0.265 |  |  |  |  | |

| **S9 Table.** Effect of parkrun community component on perceived energy | | | | |  |
| --- | --- | --- | --- | --- | --- |
| **Variable** | ***b*** | ***SE*** | ***t* (*df*)** | ***p*** | |
| Intercept | 5.657 | 0.048 | 118.36 (107.3) | < .001 | |
| Community component | 0.559 | 0.034 | 16.51 (659.5) | < .001 | |
| **Random part** | **Variance** | ***SD*** |  |  | |
| Intercept | 0.167 | 0.409 |  |  | |
| Community component | 0.582 | 0.763 |  |  | |
| $\text{R}_{\text{m}}^{\text{2}}$ = 0.295, $\text{R}_{\text{c}}^{\text{2}}$ = 0.452 |  |  |  |  | |

| **S10 Table.** Effect of who participants came or met up with on perceived energy | | | | |  |
| --- | --- | --- | --- | --- | --- |
| **Variable** | ***b*** | ***SE*** | ***t* (*df*)** | ***p*** | |
| Intercept | 5.520 | 0.082 | 67.21 (208.1) | < .001 | |
| Came or met up with family and/or friends | 0.209 | 0.081 | 2.60 (730.2) | .009 | |
| **Random part** | **Variance** | ***SD*** |  |  | |
| Intercept | 0.449 | 0.670 |  |  | |
| Came or met up with family and/or friends | 0.708 | 0.841 |  |  | |
| $\text{R}_{\text{m}}^{\text{2}}$ = 0.009, $\text{R}_{\text{c}}^{\text{2}}$ = 0.394 |  |  |  |  | |

| **S11 Table.** Effect of pre-run sociality on perceived energy | | | | |  |
| --- | --- | --- | --- | --- | --- |
| **Variable** | ***b*** | ***SE*** | ***t* (*df*)** | ***p*** | |
| Intercept | 5.436 | 0.090 | 60.16 (75.1) | < .001 | |
| Pre-run sociality | 0.310 | 0.092 | 3.38 (62.9) | .001 | |
| **Random part** | **Variance** | ***SD*** |  |  | |
| Intercept | 0.480 | 0.693 |  |  | |
| Pre-run sociality | 0.155 | 0.393 |  |  | |
| $\text{R}_{\text{m}}^{\text{2}}$ = 0.019, $\text{R}_{\text{c}}^{\text{2}}$ = 0.406 |  |  |  |  | |

| **S12 Table.** Effect of parkrun community component on subjective enjoyment | | | | |  |
| --- | --- | --- | --- | --- | --- |
| **Variable** | ***b*** | ***SE*** | ***t* (*df*)** | ***p*** | |
| Intercept | 5.709 | 0.0t56 | 101.59 (116.2) | < .001 | |
| Community component | 0.439 | 0.039 | 11.21 (673.6) | < .001 | |
| **Random part** | **Variance** | ***SD*** |  |  | |
| Intercept | 0.232 | 0.482 |  |  | |
| $\text{R}_{\text{m}}^{\text{2}}$ = 0.161, $\text{R}_{\text{c}}^{\text{2}}$ = 0.360 |  |  |  |  | |

| **S13 Table.** Effect of who participants came or met up with on subjective enjoyment | | | | |  |
| --- | --- | --- | --- | --- | --- |
| **Variable** | ***b*** | ***SE*** | ***t* (*df*)** | ***p*** | |
| Intercept | 5.574 | 0.0t81 | 68.54 (231.2) | < .001 | |
| Came or met up with family and/or friends | 0.230 | 0.086 | 2.67 (714.2) | .008 | |
| **Random part** | **Variance** | ***SD*** |  |  | |
| Intercept | 0.360 | 0.611 |  |  | |
| $\text{R}_{\text{m}}^{\text{2}}$ = 0.011, $\text{R}_{\text{c}}^{\text{2}}$ = 0.303 |  |  |  |  | |

| **S14 Table.** Effect of pre-run sociality on subjective enjoyment | | | | | | | |  |
| --- | --- | --- | --- | --- | --- | --- | --- | --- |
| **Variable** | ***b*** | ***SE*** | | | ***t* (*df*)** | ***p*** | | |
| Intercept | 5.479 | 0.0t85 | | | 65.75 (64.6) | < .001 | | |
| Pre-run sociality | 0.346 | 0.010 | | | 3.47 (80.8) | < .001 | | |
| **Random part** | **Variance** | ***SD*** | | |  |  | | |
| Intercept | 0.304 | 0.552 | | |  |  | | |
| Pre-run sociality | 0.259 | 0.509 | | |  |  | | |
| $\text{R}_{\text{m}}^{\text{2}}$ = 0.023, $\text{R}_{\text{c}}^{\text{2}}$ = 0.336 |  | |  |  | | |  | |

| **S15 Table.** Effect of parkrun community component on logged 5 km run times | | | | |  |
| --- | --- | --- | --- | --- | --- |
| **Variable** | ***b*** | ***SE*** | ***t* (*df*)** | ***p*** | |
| Intercept | 3.292 | 0.017 | 197.71 (140.7) | < .001 | |
| Community component | 0.008 | 0.040 | 1.87 (75.5) | .065 | |
| Pace influence | −0.104 | 0.008 | −12.90 (610.9) | < .001 | |
| **Random part** | **Variance** | ***SD*** |  |  | |
| Intercept | 0.038 | 0.194 |  |  | |
| Community component | < 0.001 | 0.014 |  |  | |
| $\text{R}_{\text{m}}^{\text{2}}$ = 0.037, $\text{R}_{\text{c}}^{\text{2}}$ = 0.900 |  |  |  |  | |

| **S16 Table.** Effect of who participants came or met up with on logged 5 km run times | | | | |  |
| --- | --- | --- | --- | --- | --- |
| **Variable** | ***b*** | ***SE*** | ***t* (*df*)** | ***p*** | |
| Intercept | 3.287 | 0.017 | 187.92 (135.3) | < .001 | |
| Came or met up with family and/or friends | 0.007 | 0.008 | 0.94 (75.5) | 0.354 | |
| Pace influence | −0.104 | 0.008 | −12.75 (609.8) | < .001 | |
| **Random part** | **Variance** | ***SD*** |  |  | |
| Intercept | 0.039 | 0.197 |  |  | |
| Came or met up with family and/or friends | < 0.001 | 0.028 |  |  | |
| $\text{R}_{\text{m}}^{\text{2}}$ = 0.036, $\text{R}_{\text{c}}^{\text{2}}$ = 0.900 |  |  |  |  | |

| **S17 Table.** Effect of pre-run sociality on logged 5 km run times | | | | |  |
| --- | --- | --- | --- | --- | --- |
| **Variable** | ***b*** | ***SE*** | ***t* (*df*)** | ***p*** | |
| Intercept | 3.284 | 0.017 | 190.30 (139.9) | < .001 | |
| Pre-run sociality | 0.011 | 0.008 | 1.31 (63.5) | 0.196 | |
| Pace influence | −0.103 | 0.008 | −12.67 (609.4) | < .001 | |
| **Random part** | **Variance** | ***SD*** |  |  | |
| Intercept | 0.037 | 0.193 |  |  | |
| Pre-run sociality | 0.001 | 0.034 |  |  | |
| $\text{R}_{\text{m}}^{\text{2}}$ = 0.035, $\text{R}_{\text{c}}^{\text{2}}$ = 0.901 |  |  |  |  | |

| **S18 Table.** Effect of parkrun community component on perceived energy | | | | |  |
| --- | --- | --- | --- | --- | --- |
| **Variable** | ***b*** | ***SE*** | ***t* (*df*)** | ***p*** | |
| Intercept | 5.654 | 0.047 | 120.35 (102.5) | < .001 | |
| Community component | 0.566 | 0.043 | 13.14 (103.1) | < .001 | |
| Pace influence | 0.319 | 0.080 | 3.96 (718.4) | < .001 | |
| **Random part** | **Variance** | ***SD*** |  |  | |
| Intercept | 0.141 | 0.376 |  |  | |
| Community component | 0.079 | 0.280 |  |  | |
| $\text{R}_{\text{m}}^{\text{2}}$ = 0.314, $\text{R}_{\text{c}}^{\text{2}}$ = 0.518 |  |  |  |  | |

| **S19 Table.** Effect of parkrun community component on logged 5 km run times, while controlling for perceived energy | | | | |
| --- | --- | --- | --- | --- |
| **Variable** | ***b*** | ***SE*** | ***t* (*df*)** | ***p*** |
| Intercept | 3.377 | 0.026 | 127.59 (561.9) | < .001 |
| Perceived energy | −0.015 | 0.004 | −4.15 (595.6) | < .001 |
| Community component | 0.015 | 0.004 | 3.51 (105.0) | < .001 |
| Pace influence | −0.010 | 0.008 | −12.22 (611.5) | < .001 |
| **Random part** | **Variance** | ***SD*** |  |  |
| Intercept | 0.037 | 0.193 |  |  |
| Community component | < 0.001 | 0.012 |  |  |
| $\text{R}_{\text{m}}^{\text{2}}$ = 0.040, $\text{R}_{\text{c}}^{\text{2}}$ = 0.900 |  |  |  |  |

| **S20 Table.** Effect of who participants came or met up with on perceived energy | | | | |  |
| --- | --- | --- | --- | --- | --- |
| **Variable** | ***b*** | ***SE*** | ***t* (*df*)** | ***p*** | |
| Intercept | 5.309 | 0.142 | 37.29 (553.6) | < .001 | |
| Came or met up with family and/or friends | 0.216 | 0.080 | 2.70 (728.9) | .007 | |
| Pace influence | 0.295 | 0.094 | −3.13 (670.3) | .002 | |
| **Random part** | **Variance** | ***SD*** |  |  | |
| Intercept | 0.437 | 0.661 |  |  | |
| $\text{R}_{\text{m}}^{\text{2}}$ = 0.027, $\text{R}_{\text{c}}^{\text{2}}$ = 0.394 |  |  |  |  | |

| **S21 Table.** Effect of who participants came or met up with on logged 5 km run times, while controlling for perceived energy | | | | |  |
| --- | --- | --- | --- | --- | --- |
| **Variable** | ***b*** | ***SE*** | ***t* (*df*)** | ***p*** | |
| Intercept | 3.328 | 0.027 | 121.4 (248.4) | < .001 | |
| Perceived energy | −0.009 | 0.003 | −2.75 (618.0) | .006 | |
| Came or met up with family and/or friends | 0.009 | 0.008 | 1.14 (50.1) | .260 | |
| Pace influence | −0.102 | 0.008 | −12.44 (607.5) | < .001 | |
| **Random part** | **Variance** | ***SD*** |  |  | |
| Intercept | 0.042 | 0.204 |  |  | |
| Came or met up with family and/or friends | 0.001 | 0.030 |  |  | |
| $\text{R}_{\text{m}}^{\text{2}}$ = 0.039, $\text{R}_{\text{c}}^{\text{2}}$ = 0.900 |  |  |  |  | |

| **S22 Table.** Effect of pre-run sociality on perceived energy | | | | |  |
| --- | --- | --- | --- | --- | --- |
| **Variable** | ***b*** | ***SE*** | ***t* (*df*)** | ***p*** | |
| Intercept | 5.133 | 0.162 | 31.67 (57.7) | < .001 | |
| Pre-run sociality | 0.312 | 0.089 | 3.51 (59.0) | < .001 | |
| Pace influence | 0.286 | 0.094 | 3.05 (689.4) | < .001 | |
| **Random part** | **Variance** | ***SD*** |  |  | |
| Intercept | 0.745 | 0.863 |  |  | |
| Pre-run sociality | 0.118 | 0.344 |  |  | |
| $\text{R}_{\text{m}}^{\text{2}}$ = 0.030, $\text{R}_{\text{c}}^{\text{2}}$ = 0.405 |  |  |  |  | |

| **S23 Table.** Effect of pre-run sociality on logged 5 km run times, while controlling for perceived energy | | | | |
| --- | --- | --- | --- | --- |
| **Variable** | ***b*** | ***SE*** | ***t* (*df*)** | ***p*** |
| Intercept | 3.320 | 0.027 | 124.2 (256.9) | < .001 |
| Perceived energy | −0.009 | 0.003 | −2.72 (618.2) | .007 |
| Pre-run sociality | 0.013 | 0.008 | 1.58 (62.1) | .118 |
| Pace influence | −0.101 | 0.008 | −12.38 (606.5) | < .001 |
| **Random part** | **Variance** | ***SD*** |  |  |
| Intercept | 0.037 | 0.192 |  |  |
| Pre-run sociality | 0.001 | 0.032 |  |  |
| $\text{R}_{\text{m}}^{\text{2}}$ = 0.037, $\text{R}_{\text{c}}^{\text{2}}$ = 0.902 |  |  |  |  |

| **S24 Table.** Effect of parkrun community component on subjective fatigue | | | | |  |
| --- | --- | --- | --- | --- | --- |
| **Variable** | ***b*** | ***SE*** | ***t* (*df*)** | ***p*** | |
| Intercept | 4.820 | 0.061 | 78.66 (105.4) | < .001 | |
| Community component | 0.061 | 0.055 | −1.12 (74.9) | .265 | |
| Pace influence | 1.257 | 0.114 | 11.08 (719.4) | < .001 | |
| **Random part** | **Variance** | ***SD*** |  |  | |
| Intercept | 0.211 | 0.459 |  |  | |
| Community component | 0.090 | 0.301 |  |  | |
| $\text{R}_{\text{m}}^{\text{2}}$ = 0.143, $\text{R}_{\text{c}}^{\text{2}}$ = 0.329 |  |  |  |  | |

| **S25 Table.** Effect of parkrun community component on logged 5 km run times, while controlling for subjective fatigue | | | | |  |
| --- | --- | --- | --- | --- | --- |
| **Variable** | ***b*** | ***SE*** | ***t* (*df*)** | ***p*** | |
| Intercept | 3.403 | 0.020 | 167.62 (287.5) | < .001 | |
| Fatigue | −0.023 | 0.002 | −9.62 (602.5) | < .001 | |
| Community component | 0.008 | 0.004 | 2.10 (73.5) | .039 | |
| Pace influence | −0.077 | 0.008 | −9.42 (607.8) | < .001 | |
| **Random part** | **Variance** | ***SD*** |  |  | |
| Intercept | 0.039 | 0.196 |  |  | |
| Community component | < 0.001 | 0.012 |  |  | |
| $\text{R}_{\text{m}}^{\text{2}}$ = 0.054, $\text{R}_{\text{c}}^{\text{2}}$ = 0.915 |  |  |  |  | |

| **S26 Table.** Effect of who participants came or met up with on subjective fatigue | | | | |  |
| --- | --- | --- | --- | --- | --- |
| **Variable** | ***b*** | ***SE*** | ***t* (*df*)** | ***p*** | |
| Intercept | 5.100 | 0.154 | 33.21 (85.9) | < .001 | |
| Came or met up with family and/or friends | −0.200 | 0.102 | −1.94 (91.7) | .054 | |
| Pace influence | 1.269 | 0.116 | 10.92 (704.9) | < .001 | |
| **Random part** | **Variance** | ***SD*** |  |  | |
| Intercept | 0.298 | 0.546 |  |  | |
| Came or met up with family and/or friends | 0.245 | 0.495 |  |  | |
| $\text{R}_{\text{m}}^{\text{2}}$ = 0.149, $\text{R}_{\text{c}}^{\text{2}}$ = 0.342 |  |  |  |  | |

| **S27 Table.** Effect of who participants came or met up with on logged 5 km run times, while controlling for subjective fatigue | | | | | |  |
| --- | --- | --- | --- | --- | --- | --- |
| **Variable** | ***b*** | ***SE*** | ***t* (*df*)** | ***p*** | | |
| Intercept | 3.402 | 0.027 | 139.17 (196.3) | < .001 | | |
| Fatigue | −0.023 | 0.002 | −9.54 (604.5) | < .001 | | |
| Came or met up with family and/or friends | < 0.001 | 0.007 | 0.04 (52.9) | .968 | | |
| Pace influence | −0.077 | 0.008 | −9.36 (602.7) | < .001 | | |
| **Random part** | **Variance** | ***SD*** |  |  | | |
| Intercept | 0.043 | 0.208 |  |  | | |
| Came or met up with family and/or friends | < 0.001 | 0.022 |  |  | | |
| $\text{R}_{\text{m}}^{\text{2}}$ = 0.053, $\text{R}_{\text{c}}^{\text{2}}$ = 0.914 |  |  |  |  | | |
| **S28 Table.** Effect of pre-run sociality on subjective fatigue | | | | |  |  |
| **Variable** | ***b*** | ***SE*** | ***t* (*df*)** | ***p*** | | |
| Intercept | 4.860 | 0.168 | 28.85 (556.2) | < .001 | | |
| Pre-run sociality | −0.028 | 0.095 | −0. 289 (683.6) | .773 | | |
| Pace influence | 1.286 | 0.115 | 11.21 (730.8) | < .001 | | |
| **Random part** | **Variance** | ***SD*** |  |  | | |
| Intercept | 0.251 | 0.501 |  |  | | |
| Pre-run sociality | 1.138 | 1.067 |  |  | | |
| $\text{R}_{\text{m}}^{\text{2}}$ = 0.145, $\text{R}_{\text{c}}^{\text{2}}$ = 0.300 |  |  |  |  | | |

| **S29 Table.** Effect of pre-run sociality on logged 5 km run times, while controlling for subjective fatigue | | | | |  |
| --- | --- | --- | --- | --- | --- |
| **Variable** | ***b*** | ***SE*** | ***t* (*df*)** | ***p*** | |
| Intercept | 3.390 | 0.023 | 146.66 (419.3) | < .001 | |
| Subjective fatigue | −0.023 | 0.002 | −9.55 (605.4) | < .001 | |
| Pre-run sociality | 0.007 | 0.007 | 1.10 (610.6) | .271 | |
| Pace influence | −0.077 | 0.008 | −9.39 (609.9) | < .001 | |
| **Random part** | **Variance** | ***SD*** |  |  | |
| Intercept | 0.039 | 0.196 |  |  | |
| $\text{R}_{\text{m}}^{\text{2}}$ = 0.054, $\text{R}_{\text{c}}^{\text{2}}$ = 0.915 |  |  |  |  | |

| **S30 Table.** Effect of parkrun community component on subjective effort (RPE) | | | | |  |
| --- | --- | --- | --- | --- | --- |
| **Variable** | ***b*** | ***SE*** | ***t* (*df*)** | ***p*** | |
| Intercept | 9.652 | 0.151 | 63.98 (115.7) | < .001 | |
| Community component | 0.135 | 0.094 | 1.45 (712.0) | .149 | |
| **Random part** | **Variance** | ***SD*** |  |  | |
| Intercept | 2.048 | 1.431 |  |  | |
| $\text{R}_{\text{m}}^{\text{2}}$ = 0.003, $\text{R}_{\text{c}}^{\text{2}}$ = 0.338 |  |  |  |  | |

| **S31 Table.** Effect of who participants came or met up with on subjective effort (RPE) | | | | |  |
| --- | --- | --- | --- | --- | --- |
| **Variable** | ***b*** | ***SE*** | ***t* (*df*)** | ***p*** | |
| Intercept | 9.798 | 0.161 | 60.92 (87.7) | < .001 | |
| Came or met up with family and/or friends | −0.278 | 0.205 | −1.36 (609.9) | .178 | |
| **Random part** | **Variance** | ***SD*** |  |  | |
| Intercept | 1.240 | 1.113 |  |  | |
| Came or met up with family and/or friends | 0.882 | 0.939 |  |  | |
| $\text{R}_{\text{m}}^{\text{2}}$ = 0.031, $\text{R}_{\text{c}}^{\text{2}}$ = 0.368 |  |  |  |  | |

| **S32 Table.** Effect of pre-run sociality on subjective effort (RPE) | | | | |  |
| --- | --- | --- | --- | --- | --- |
| **Variable** | ***b*** | ***SE*** | ***t* (*df*)** | ***p*** | |
| Intercept | 9.708 | 0.196 | 49.57 (247.9) | < .001 | |
| Pre-run sociality | 0.135 | 0.094 | 1.45 (712.0) | .149 | |
| **Random part** | **Variance** | ***SD*** |  |  | |
| Intercept | −0.094 | 0.190 |  |  | |
| $\text{R}_{\text{m}}^{\text{2}}$ < 0.001, $\text{R}_{\text{c}}^{\text{2}}$ = 0.354 |  |  |  |  | |

**References**

1. Stevinson C, Wiltshire G, Hickson M. Facilitating participation in health-enhancing physical activity: A qualitative study of parkrun. International Journal of Behavioral Medicine. 2014;22(2):170 - 7.

2. Hindley D. “More than just a run in the park”: An exploration of parkrun as a shared leisure space. Leisure Sciences. 2018;42(1):1-21.

3. Stevinson C, Hickson M. Exploring the public health potential of a mass community participation event. Journal of Public Health. 2014;36(2):268-74.

4. Wiltshire G, Stevinson C. Exploring the role of social capital in community-based physical activity: Qualitative insights from parkrun. Qualitative Research in Sport, Exercise and Health. 2017;10(1):1-16.

5. Morris P, Scott H. Not just a run in the park: A qualitative exploration of parkrun and mental health. Advances in Mental Health. 2018;17(2):1-14.

6. Barr DJ, Levy R, Scheepers C, Tily HJ. Random effects structure for confirmatory hypothesis testing: Keep it maximal. Journal of Memory and Language. 2013;68(3):255-78.

7. Bell BA, Ferron JM, Kromrey JD, editors. Cluster size in multilevel models: The impact of sparse data structures on point and interval estimates in two-level models. Proceedings of the Joint Statistical Meetings; 2008.

8. Benjamini Y, Yekutieli D. The control of the false discovery rate in multiple testing under dependency. The Annals of Statistics. 2001;29(4):1165-88.

9. Benjamini Y, Hochberg Y. Controlling the false discovery rate: A practical and powerful approach to multiple testing. Journal of the Royal Statistical Society: Series B (Methodological). 1995;57(1):289-300.

10. Cribbie RA. Multiplicity control in structural equation modeling. Structural Equation Modeling. 2007;14(1):98-112.

11. Hayes AF. Introduction to mediation, moderation, and conditional process analysis. London: The Guilford Press; 2013.

12. Jiang H, Eskridge KM, editors. Bias in principal components analysis due to correlated observations. Conference on Applied Statistics in Agriculture; 2000.

13. Snijders TAB, Bosker RJ. Multilevel analysis: An introduction to basic and advanced multilevel modeling. 2nd ed: Sage; 2012.

14. Raudenbush SW, Bryk AS. Hierarchical linear models: Applications and data analysis methods: Sage; 2002.

15. Imai K, Keele L, Yamamoto T. Identification, inference and sensitivity analysis for causal mediation effects. Statistical Science. 2010;25(1):51-71.

16. Seco GV, García MA, García MPF, Rojas PEL. Multilevel bootstrap analysis with assumptions violated. Psicothema. 2013;25(4):520-8.

17. Maas CJ, Hox JJ. Sufficient sample sizes for multilevel modeling. Methodology. 2005;1(3):86-92.

18. Maas CJ, Hox JJ. Robustness issues in multilevel regression analysis. Statistica Neerlandica. 2004;58(2):127-37.

19. Tingley D, Yamamoto T, Hirose K, Keele L, Imai K. Mediation: R package for causal mediation analysis. 2014.
